# Saponin and Fatty Acid Profiling of the Sea Cucumber *Holothuria atra*, α-Glucosidase Inhibitory Activity and the Identification of a Novel Triterpene Glycoside

**DOI:** 10.3390/nu15041033

**Published:** 2023-02-19

**Authors:** Yunita Eka Puspitasari, Emmy Tuenter, Kenn Foubert, Herawati Herawati, Anik Martinah Hariati, Aulanni’am Aulanni’am, Luc Pieters, Tess De Bruyne, Nina Hermans

**Affiliations:** 1 Natural Products and Food Research & Analysis—Pharmaceutical Technology (NatuRAPT), University of Antwerp, 2610 Antwerpen, Belgium; 2 Department of Fish Product Technology, Faculty of Fisheries and Marine Sciences, Universitas Brawijaya, Malang 65149, Indonesia; 3 Doctoral Program of Environmental Studies, Postgraduate School, Universitas Brawijaya, Malang 65145, Indonesia; 4 Faculty of Veterinary Medicine, Universitas Brawijya, Malang 65145, Indonesia; 5 Department of Aquaculture, Faculty of Fisheries and Marine Sciences, Universitas Brawijaya, Malang 65145, Indonesia; 6 Biochemistry Laboratory, Faculty of Sciences, Universitas Brawijaya, Malang 65145, Indonesia

**Keywords:** *Holothuroidea*, triterpene glycosides, *Holothuria*, α-glucosidase

## Abstract

Saponin-rich sea cucumber extracts have shown antidiabetic effects in a few reports. Although the triterpene glycosides of sea cucumbers are commonly isolated from their Cuvierian tubules, these are absent in *Holothuria atra* Jaeger. Therefore, this study intended to investigate the saponin profile in the body wall of *H. atra*, as well as to assess the α-glucosidase inhibitory activity of the *H. atra* extracts. The chemical profiling of sea cucumber extracts was conducted by UPLC-HRMS analysis. This resulted in the tentative identification of 11 compounds, 7 of which have not been reported in the *H. Atra* body wall before. Additionally, two triterpene glycosides were purified and their structures were elucidated based on HRMS and NMR data: desholothurin B (1), and a novel epimer, 12-epi-desholothurin B (2). Moreover, the fatty acid profile of the *H. atra* body wall was investigated by GC-MS. It was found that the Me90 fraction of the *H. atra* body wall showed the strongest α-glucosidase inhibitory activity (IC_50_ value 0.158 ± 0.002 mg/mL), thus making it more potent than acarbose (IC_50_ value 2.340 ± 0.044 mg/mL).

## 1. Introduction

Sea cucumbers (*Holothuroidea*) belong to the marine invertebrate Echinoderms. They are distributed in benthic areas, deep seas, and also in coral reefs. In traditional Chinese medicine, sea cucumbers are consumed for their beneficial health properties and are considered one of the delicacies of Asian-Pacific cuisine [1,2]. The traditional use of sea cucumbers may be linked to the presence of saponins, which were reported to possess antifungal [3,4], antiviral [5], antioxidant [6], and cytotoxicity activities [7,8,9,10].

Various species of sea cucumbers, including some *Holothuria* sp., have Cuvierian tubules, a gland expelled from the anus to defend the organism from marine animal attacks. The Cuvierian tubules contain a large variety of saponins, which are utilized as a chemical defense [11]. In addition, saponins can also be found in other parts of the sea cucumber, including the body wall and viscera [11,12], as well as in the seawater surrounding the sea cucumbers [13].

Thus, apart from higher plants [14], marine organisms such as sea cucumbers [15,16,17], sea stars [18,19,20,21], and sponges [22,23,24] can contain saponins. Saponins are glycosidic secondary metabolites consisting of either a triterpene (C30) or a steroid (C27) aglycone linked to a sugar moiety [25]. The saponins present in sea cucumbers are triterpene glycosides with a carbohydrate chain of two to six sugar units, composed of D-xylose (Xyl), D-quinovose (Qui), 3-*O*-methyl-D-glucose (MeGlc), D-glucose (Glc) [26], 3-*O*-methyl-xylose (MeXyl) [27], and infrequently, 3-*O*-methyl-D-quinovose [28,29]. The saponins in sea cucumbers are always glycosylated at position C-3 of the aglycone and C-1 of the first sugar unit, which is always xylose. In addition, MeGlc and MeXyl are always found as terminal sugars [26,30]. Moreover, the glycosides in sea cucumber saponins can be either sulfated or non-sulfated. More specifically, sulfate groups are encountered at C-4 of xylose [31]. Almost 135 saponins of the genus *Holothuria* have been reported after structure elucidation, by means of mass spectrometry (MS) and/or nuclear magnetic resonance (NMR) spectroscopy [32].

*Holothuria atra*, also called the black sea cucumber, is a marine animal that usually lives in lagoons, rocky reefs, or mudflats, and associates with seagrass [33,34]. Unlike most species of the *Holothuria* genus, *H. atra* does not possess Cuvierian tubules, but its body wall does contain saponins [12]. The prior research of Kobayashi et al. [35] and Van Dyck et al. [12] showed that *H. atra* contained only sulfated triterpene glycosides, such as echinoside A, echinoside B, and holothurins B/B4, B1, B2, and B3. In addition, 24-dehydroechinoside A was also identified in the body wall of *H. atra* [36]. Other saponins were also identified from *H. atra* calcigeroside B, holothurin A, holuthurin D, holothurinogenin B, holothurinoside K1 [37], and desulfated echinoside B [38]. Holothurin A5 was also isolated from *H. atra* [37].

Saponins have been shown to posses many physiological functions, including anti-hyperglycemia activity. The extract of sea cucumbers prepared from *Stichopus hermanii* and *S. horrens* are traditionally used in Malaysia to control the blood glucose levels in diabetic patients [39,40]. Moreover, holothurin A and echinoside A inhibited the α-glucosidase activity, resulting in suppressed postprandial blood glucose levels in the treatment of diabetic mice [41]. The administration of the saponin-containing *n*-BuOH fraction of the sea cucumber *H. thomasi* was seen to elevate the serum insulin levels and decrease the serum glucose in STZ-induced diabetic rats, and to inhibit their α-amylase activity, thus indicating that the saponins of *H. thomasi* show anti-diabetic potential [42].

Apart from saponins, sea cucumbers also contain fatty acids, as previously reported for *Stichopus japonicus* [43,44], *S. chloronatus* [45], *H. scabra, H. leucospilota, S. horrens,* and *H. atra* [46]. The fatty acids purified from the body wall of *S. japonicus* (including 7(*Z*)-octadecenoic acid and 7(*Z*), 10(*Z*)-octadecadienoic acid) and internal organs (1,3-dipalmitolein and *cis*-9-octadecenoic acid) showed strong α-glucosidase inhibitory activity [43,44].

α-glucosidase is involved in carbohydrate digestion by hydrolyzing starch into glucose. The glucose is then absorbed and transported into the bloodstream via glucose transporters. Rapidly digested and absorbed glucose in the human intestine causes an increasing blood glucose level. Inhibiting α-glucosidase activity is a possible strategy to control these blood glucose levels by slowing the carbohydrate hydrolysis and glucose absorption [47,48]. The administration of oral α-glucosidase inhibitory drugs such as acarbose, miglitol, and voglibose in diabetes management has side effects, namely diarrhea and flatulence. Therefore, investigations into a new α-glucosidase inhibitor with less side effects have been conducted, with both terrestrial and marine organisms.

The black sea cucumbers *H. atra* are found abundantly in Indonesia, but they have lower economic value compared to other sea cucumbers because of the limited information on their nutritional and medicinal value [49]. Some studies have reported the anti-diabetic potential of saponins [41,42,50,51], but the α-glucosidase inhibitory activity of saponins from *H. atra* has not been evaluated yet. Therefore, this work aimed to study the saponin profile of *H. atra*, as well as to assess the α-glucosidase inhibitory activity of *H. atra* extracts. Moreover, the fatty acid profile of the body wall of *H. atra* and its α-glucosidase inhibitory activity were studied. We hypothesized that sea cucumber saponins and fatty acids from the body wall of *H. atra* may have α-glucosidase inhibitory activity.

## 2. Materials and Methods

### 2.1. Materials

#### General Experimental Procedures

All solvents, including dichloromethane (≥99.8%), methanol (≥99.8%), *n*-hexane (95%), acetonitrile (≥99.8%), propan-2-ol (≥99.5%), *n*-butanol (≥99%), and ethyl acetate (99.5%) were purchased from Acros Organics (Thermo Fisher Scientific Inc, Geel, Belgium) and Fisher Scientific (Thermo Fisher Scientific Inc, Loughborough, United Kingdom). Acetonitrile and formic acid with UPLC-grade were purchased from Biosolve (Biosolve BV, Valkenswaard, the Netherlands). Formic acid (98%), sulphuric acid (≥95%), dimethylsulfoxide (DMSO), and glacial acetic acid (99.8%) were obtained from Acros Organics (Thermo Fisher Scientific Inc, Geel, Belgium) and Fisher Scientific (Thermo Fisher Scientific Inc, Loughborough, United Kingdom). Deuterated solvents, including methanol-*d*_4_ and pyridine-*d*_5_, were obtained from Sigma Aldrich (Sigma-Aldrich Chemie GmbH, Steinheim, Germany). Ultra-pure water was dispensed by a Milli-Q system (Rephile Bioscience Ltd, Belgium) and was passed through a 0.2 μm membrane filter.

The materials used for the α-glucosidase assay: α-glucosidase from *Saccharomyces cerevisiae*, *p*-nitrophenyl-α-D-glucopyranoside (PNPG), and acarbose (≥95%) were purchased from Sigma-Aldrich (Sigma-Aldrich Chemie GmbH, Steinheim, Germany); the 96-well plates were from Thermo Scientific Nunc (Thermo Fisher Scientific Inc., Roskilde, Denmark) and the Na_2_HPO_4_·2H_2_O was from Merck (MerckKGaA, Darmstadt, Germany). The reference standard for the GC-MS analysis was a Supelco 37 Component FAME mix from Sigma Aldrich (Sigma-Aldrich Chemie GmbH, Steinheim, Germany). Pure fatty acids, including palmitoleic acid (≥98.5%), arachidonic acid, and eicosapentaenoic acid (≥99%), were purchased from Sigma Aldrich (Sigma-Aldrich Chemie GmbH, Steinheim, Germany).

### 2.2. Methods

#### 2.2.1. Sea Cucumber Material

Fresh *H. atra* sea cucumbers (12 kg) were collected in October 2016 from East Java, Indonesia. The fresh sea cucumbers were approximately 20 cm in length and 200 g in weight. Their identification was confirmed by the Research Center for Oceanography, Indonesian Institute of Sciences (voucher number B-173/IPK.2/IF/I/2017). The sea cucumbers were kept in a coolbox packed with ice. They were eviscerated to separate the body wall from their internal organs and washed with water. The body walls (3 kg) were dried in open air (±24 °C for 5 days, being exposed to direct sunlight for approximately eight hours a day), and were milled and sieved (sieve width 40 mm) to obtain a fine powder (600 g) using a disk mill machine. The moisture content of the dried *H. atra* was 9% and was determined using the drying method. The measurement was carried out by drying the samples in an oven at 105 °C until the constant weight was reached [52].

#### 2.2.2. Preparation of Sea Cucumber *H. atra* Extract

**Figure 1:**
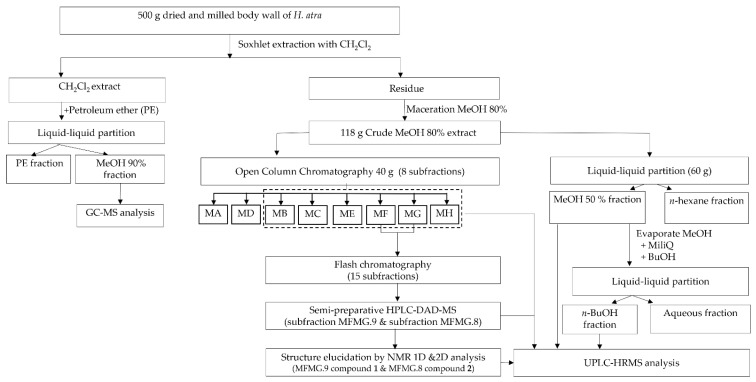
General scheme of extraction, fractionation, and chemical profiling of the *H. atra* body wall extract.

The preparation of the sea cucumber extract was carried out according to Sottorf et al. (2013), with minor modifications [53]. An extract was prepared from the dried and milled material (500 g) using a soxhlet apparatus, and 6 L of CH_2_Cl_2_ and 6.5 g of CH_2_Cl_2_ extract (DCM) were resuspended in MeOH 90% (Me90), and then partitioned with 200 mL petroleum ether. Both subfractions were dried under reduced pressure at 40 °C. The residue after the initial soxhlet extraction was macerated with 13 L of MeOH 80%, and it was concentrated under reduced pressure and freeze-dried to obtain a methanolic crude extract (118 g). Figure 1 summarizes the general procedure of the extraction, fractionation, and chemical proflling of the sea cucumber *H. atra.*

#### 2.2.3. Fractionation of Triterpene Glycosides

The fractionation of the triterpene glycosides was carried out by means of a liquid–liquid partition. In this procedure, the methanolic crude extract (60 g) was subjected to the liquid–liquid partition. It was solubilized in MeOH 50% (600 mL) and partitioned against *n*-hexane (600 mL). The *n*-hexane fraction and MeOH 50% fraction were concentrated under reduced pressure to obtain Hex fraction and Me fraction. The Me fraction was redissolved in 600 mL of water and was partitioned against 600 mL of *n*-BuOH. Both the Me fraction and the *n*-BuOH fraction (Bu fraction) were concentrated under reduced pressure and freeze-dried. The Me fraction and Bu fraction were submitted to UPLC-HRMS. The fractionation was carried out using open column chromatography and flash chromatography. The flash chromatography was performed on a Reveleris iES system from Grace (Columbia, MD, USA), with Reveleris^®^ Navigator™ software.

#### 2.2.4. Isolation of Triterpene Glycosides

The methanolic extract (40 g) was subjected to open column chromatography (column dimensions 100 × 1000 mm), with Diaion HP 20 as stationary phase, starting with 100% water as an eluting solvent followed by water:MeOH (6:4), gradually changing to 100% MeOH. All the fractions were dried under reduced pressure. This resulted in the following subfractions: 100% H_2_O (MA), MeOH 40% (MB), MeOH 50% (MC), MeOH 60% (MD), MeOH 70% (ME), MeOH 80% (MF), MeOH 90% (MG), and MeOH 100% (MH). The subfractions MB, MC, ME, MF, and MG were submitted to UPLC-HRMS. The subfractions MA and MD were not submitted to UPLC-HRMS, since those subfractions did not reveal any spots after NP-TLC analysis. The subfractions MF and MG were combined because they showed a similar pattern after NP-TLC analysis.

The subfraction MF/MG (2.3653 mg) was submitted to flash chromatography on a Reveleris C_18_ cartridge by solid injection. The mobile phase used was H_2_O + 0.1% formic acid (A) and acetonitrile + 0.1% formic acid (B), with the following gradient: 0 min 95% for A and 5% of B retained for 15 min, which then changed to 50% for A and 50% for B at 52 min; from 97–111 min, a linear change to 0% for A and 100% for B, and from 111 min, a linear change to 95% for A and 5% for B, with a flow rate of 13 mL/min. The eluent was collected according to the signals measured by the evaporative light scattering detector (ELSD) and ultraviolet (UV) absorption at 210 and 254 nm; NP-TLC analysis was performed with *n*-BuOH-AcOH-H_2_O 4-1-5 as the mobile phase. The subfractions of FG showing a similar pattern were combined, resulting in 15 subfractions.

Isolation was conducted on a semi-preparative HPLC-DAD-MS system (Waters, Millford, MA, USA) with Masslynx™software version 4.1. A Phenomenex Luna C18(2) 100 Å; 250 × 10.00 mm, 5 μm column was used together with a pre-column. The subfraction MFMG.9 (101.9 mg) was further purified by semi-preparative HPLC-DAD-MS with a C18 Luna column and the mobile phase H_2_O + 0.1% formic acid (A) and acetonitrile + 0.1% formic acid (B), and the following gradient: 0 to 5 min 42% of B, 30 min 50% of B, 35–40 min 100% of B, and 45–55 42% of B. The flow rate was 4.75 mL/min. The mass spectrometer was operated in ESI+ mode, with an MS scan range of *m*/*z* 250 to 800; V_capillary_ 3.5 kV; V_cone_ 50 V; V_extractor_ 3V; V_RF Lens_ 0.2 V; T_source_ 125 °C; T_desolvation_ 400 °C, and a desolvation gas flow of 750 L/h and a cone gas flow of 0 L/h to collect a compound with *m*/*z* 485.2 and *m*/*z* 763.3 (compound **1**).

The subfraction MFMG.8 (175 mg) was submitted to the semi-preparative HPLC-DAD-MS with the following gradient: 0 to 5 min 35% of B, 10 min 50% of B, 35–40 min 100% of B, and 45–55 32% of B, to collect a compound with *m*/*z* 485.2 and *m*/*z* 763.3 (compound **2**).

#### 2.2.5. Structure Elucidation Using 1D and 2D NMR

A Bruker DRX-400 instrument (Rheinstetten, Germany) was used to record NMR spectra and was equipped with a 3 mm broadband inverse (BBI) probe or a 5 mm dual ^1^H/^13^C probe. Standard Bruker pulse sequences were used to record ^1^H, ^13^C, DEPT135, DEPT90, and 2D (COSY, HSQC, HMBC, NOESY) NMR spectra. Bruker TopSpin version 4.0.8 for Windows (Billerica, MA, USA) was used to process the NMR data.

Desholothurin B (1): white powder (1.6 mg); ^1^H NMR (methanol-d_4_); and ^13^C NMR (methanol-d_4_): HRMS *m*/*z* 803.4250 [M+Na]^+^ (calcd for C_41_H_64_O_14_Na: 803.4194; and *m*/*z* 485.3267 (aglycon).

12-epi-Desholothurin B (2): white powder (1.9 mg); ^1^H NMR (pyridine-d_5_); and ^13^C NMR (pyridine-d_5_): HRMS 803.4175 [M+Na]^+^ (calcd for C_41_H_64_O_14_Na: 803.4194; and *m*/*z* 485.3256 (aglycon).

#### 2.2.6. UPLC-HRMS Analysis

UPLC-HRMS analysis of the *H. atra* extracts was carried out according to Rivera-Mondragon et al. (2019), with modifications [54], using a XEVO-G2-XS QTOF mass spectrometer (Waters, Milford, MA, USA) coupled with an Acquity UPLC system. The system was operated with MassLynx 4.1 software (Waters, Milford, MA, USA). An HSS T3 RP18 column (1.8 µm, 2.1 × 100 mm) (Waters, Milford, MA, USA) was used to obtain separation. The following samples were analyzed using a UPLC Acquity system coupled with a Xevo G2-XS Q-Tof mass spectrometer (Waters, Milford, MA, USA), including subfractions MB, MC, ME, MF, and MG, and the Me fraction and Bu fraction. A total of two isolated compounds were also analyzed by UPLC-HRMS. The mobile phases used were (A) H_2_O + 0.1% FA and (B) ACN + 0.1% FA, and the gradient was set as follows: 3% of B (0–1 min), 100% of B (17–19 min), and 3% of B (21–25 min). The flow rate was 0.4 mL/min. The following settings were used for the mass spectrometer: a cone gas flow of 50 L/h; a desolvation gas flow of 1000 L/h; a source temperature of 120 °C; and desolvation at 550 °C. The samples were analyzed in MSe mode, thus obtaining information from the molecular ions and mass fragmentation data simultaneously. The MS data were recorded in ESI+ and ESI- mode with an MS scan range from *m*/*z* 50 to 1500.

#### 2.2.7. GC-MS Analysis

The GC-MS analysis was conducted according to Dendooven et al. (2021), with minor modifications [55]. The petroleum ether (PE) fraction and MeOH 90% fraction were submitted to GC-MS analysis. The GC-MS analysis was carried out using a Trace GC Ultra (Thermo Fisher Scientific, Waltham, MA, USA), equipped with a capillary column (Restex Rxi5HT (30 m × 0.25 mm, 0.25 µm film thickness) (Chrom Tech, Apple Valley, MN, USA)). A 100 µL mix of fatty acid methyl esters containing 37 compounds was analyzed simultaneously, as is the reference standard. Helium (Praxair Technology, Danbury, CT, USA) was used as carrier gas with a flow rate of 0.1 mL/min. The splitless injection volume was 1 µL, and the inlet was heated to 250 °C. The oven temperature program was set as stated: 3 min of isothermal at 170 °C, increasing the temperature by 3 °C/min to 230 °C, and finishing with 15 min of isothermal at 230 °C. The MS analysis was performed with a DSQ mass spectrometer (Thermo Fisher Scientific, Waltham, MA, USA) in scan mode within an *m*/*z* range of 50–1000 µ, with a run time of 41.75 min and scanning starting after 6 min. The GC-MS temperature of the ion source was 250 °C.

#### 2.2.8. α-Glucosidase Inhibition Assay

The α-glucosidase inhibition was assessed using the method described by Su et al. (2013 and Trinh et al. (2016), with a slight modification [56,57]. In total, 100 μL of the α-glucosidase enzyme solution from *Saccharomyces cerevisiae* (0.2 U/mL in a 0.1 M phosphate buffer at pH 6.8) and 50 μL of the sample dissolved in the phosphate buffer containing 6% DMSO 0.25–20 mg/mL), and were incubated in a transparent 96-well plate at 37 °C for 15 min. Next, 50 μL of PNPG (5 mM in a 0.1 m phosphate buffer) was added. The PNPG was hydrolyzed to release *p*-nitrophenyl (PNP), and this process was monitored at 405 nm for 30 min at 37 °C, with a BioTek Eon microplate reader (Winooski, VT, USA)) using the Gen5 version 2.06 software. The blank sample was treated in a similar way, but contained a 0.1 M phosphate buffer at pH 6.8 (containing DMSO 6%) instead of a test compound. Acarbose (≥95%, Sigma Aldrich) was used as a positive control, and all the measurements were repeated three times. The IC_50_ values were calculated with GraphPad Prism 6 software (GraphPad Software Inc, La Jolla, CA, USA). The IC_50_ values were subjected to one-way analysis of variance (ANOVA) and the Tukey post hoc test, using GraphPad Prism 6 software to assess any significant differences among the treatments. *P*-values < 0.05 were considered significant.

## 3. Results

### 3.1. Tentative Identification of Triterpene Glycosides from H. atra Body Wall

**Figure 2:**
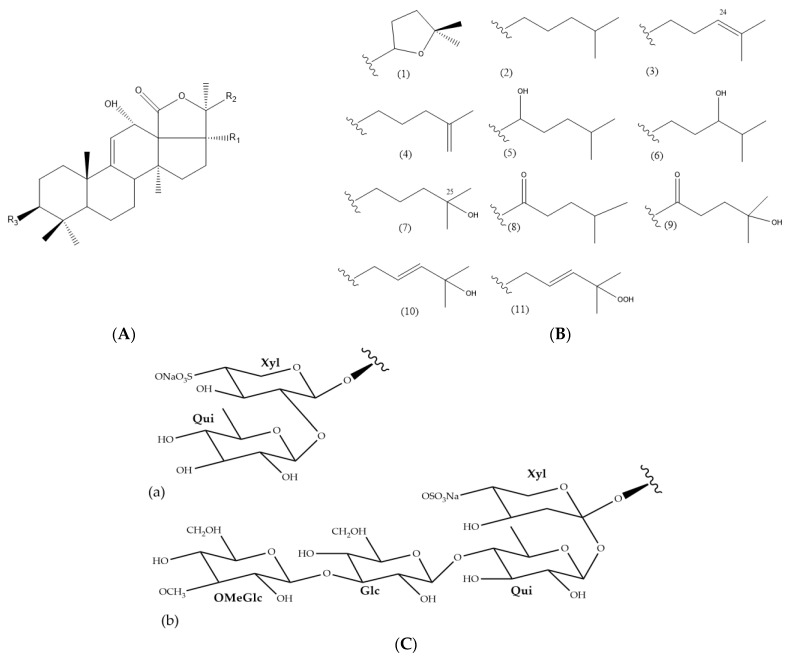
Sapogenin (**A**), sapogenin side chains (**B**/R2), and glycosidic moieties (**C**/R3) of triterpene glycosides identified in *H. atra* body wall by means of UPLC-HRMS analysis. Correspond-ing substructures of sapogenin (1–11), (a, b).

**Figure 3:**
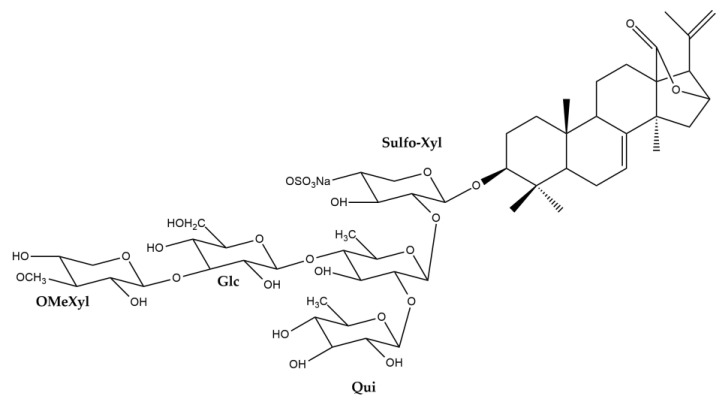
Structure of calcigeroside B.

**Figure 4:**
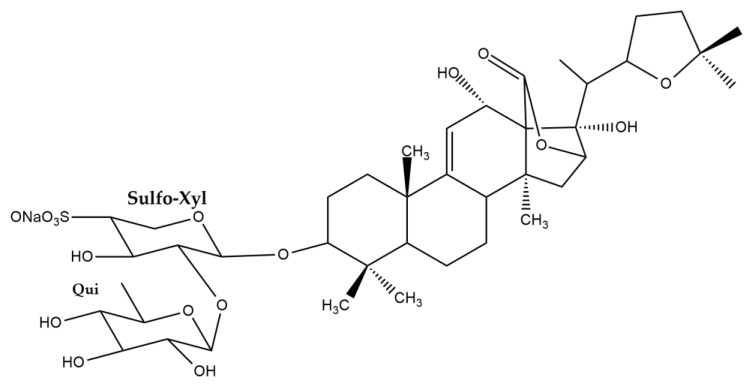
Structure of nobiliside II (=ananaside C).

The UPLC-HRMS analysis of the *H. atra* body wall extracts led to the tentative identification of triterpene glycosides in ESI^-^ mode (Table 1). The molecular structure of triterpene glycoside identified from *H. atra* body wall is given in Table 2. The types of sapogenin, sapogenin side chains and also glycosidic moieties of triterpene glycosides from *H. atra* bodywall are shown in Figure 2. Atypical structure of sapogenin from triterpene glycoside identified from *H. atra* body wall including Calcigeroside B and Nobiliside II (=ananaside C) are shown in Figure 3 and Figure 4, respectively.

### 3.2. Purified Saponins from H. atra Body Wall

A total of two compounds were isolated from the combined MF/MG subfraction. These two subfractions were combined because they showed a similar pattern after NP-TLC analysis. Subsequently, the combined fraction was submitted to RP flash chromatography, and the fractions with similar NP-TLC profiles were again combined, resulting in 15 subfractions. Subfractions 8 and 9 were selected for the semi-preparative HPLC-DAD-MS, which led to the isolation of two isomeric compounds.

**Figure 5:**
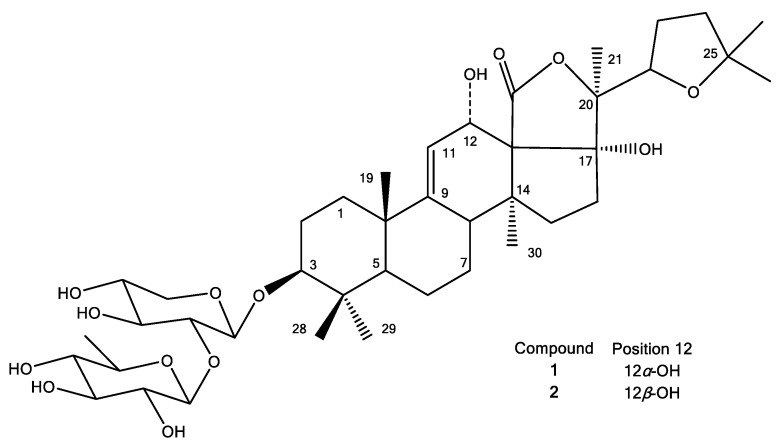
Structures of compounds **1** and **2**.

The structure elucidation of compound **1** was performed based on 1D and 2D-NMR analysis (spectra are provided as supplementary material, Figures S1–S8), as well as on UPLC-HRMS measurement (mass spectrum provided as supplementary material, Figure S14), leading to its identification as desholothurin B (desulfated holothurin B) (Figure 5, Table 3). Indeed, the ^13^C chemical shifts of compound **1** were in agreement with those previously reported [84,85] for the desulfated derivative of holothurin B, which was derived from holothurin B by means of refluxing in dioxane/pyridine (1:1). Kobayashi and co-workers obtained the desulfated holothurin B from holothurin B in a similar way [11]. Thus, in these studies, desulfated holothurin B was not obtained as natural product but as derivative of holothurin B. However, in 1987, Oleinikova and Kuznetsova had already studied the glycosidic fraction of *H. atra* and reported that this fraction contained 2.15% of desulfated holothurin B, while holothurin B was found to be the major constituent (84.2%) [86]. Unfortunately, no experimental data regarding this identification were provided, but nevertheless, this publication must be considered the first and only publication thus far to report the presence of desholothurin B as natural product. In addition, a second compound was isolated, which showed the same *m/z*-value in UPLC-HRMS, but a different retention time. Based on the result of UPLC-HRMS analysis, compounds **1** and **2** showed *m*/*z* values of 803.4250 [M+Na]^+^ and 803.4175 [M+Na]^+^, respectively, both corresponding with the molecular formula C_41_H_64_O_14_Na. The fragment ions of compounds **1** and **2** were found at *m*/*z* values 485.3275 and 485.3256, respectively, corresponding to the aglycone moieties (supplementary material, Figures S14–S17). Furthermore, also noteworthy was a difference in solubility that was noticed when preparing the compounds for NMR analysis: while compound **1** was soluble in methanol-*d*_4_, this was not the case for compound **2,** and the latter was therefore analyzed in pyridine-*d*_5_. Given the low amount of sample available, unfortunately no decent ^13^C- and DEPT-spectra could be recorded for compound **2**. Nevertheless, a complete assignment could be performed based on the ^1^H- and 2D-spectra (supplementary material, Figures S9–S13), with the exception of C-18.

The majority of ^13^C-NMR signals observed for compound **2** were similar to those of compound **1** (Table 3), and could be assigned by comparison with the NMR assignment of compound **1**, in combination with the interpretation of the 2D-NMR spectra. However, six out of thirty of the ^13^C chemical shifts showed a difference >2 ppm compared to the signals of **1**, while the other signals typically differed less than 1 ppm, again indicating a structural difference between the two compounds. These ^13^C-NMR signals were assigned to C-9 and C-11–C-14 of ring C, as well as C-17 of the holostane sapogenin moiety as follows: firstly, a signal at 151.2 ppm showed a cross peak in the HMBC-spectrum with the ^1^H-signal of the methylgroup in postion 19, and was assigned to C-9, since all other signals in close proximity to C-19 were already assigned. A total of two rather downfield ^1^H-signals, found at 5.62 and 5.25 ppm, showed a COSY interaction and were found to correspond to positions 11 and 12. Their exact positions were deduced after the assignment of the ^13^C-signals of C-11 and C-12: the ^13^C-signal at 119.5 ppm complies with a methine substructure, as in position 11, while the ^13^C-signal at 66.9 ppm complies with a hydroxylated tertiary carbon. Therefore, based on the HSQC-spectrum, the signals at 5.62 and 5.25 ppm were assigned to H-9 and H-11, respectively. The two quaternary carbon signals, C-13 and C-14, at 65.3 and 48.7 ppm, respectively, both showed a cross peak in the HMBC-spectrum, with the ^1^H-signal of the methylgroup in position 30 (1.37 ppm), thus supporting their presence in these positions. Given the proximity of the hydroxyl group in position 12, and the carbonyl-group in position 18, C-13 was assigned the more downfield signal. A ^13^C-signal, differing from the signals observed for compound **1,** remained: a quaternary carbon at 87.4 ppm. This signal was assigned to position C-17, which was supported by an HMBC-correlation with the ^1^H-signal of the methyl group in position 21 (2.04 ppm).

The highest discrepancy of the chemical shifts observed for the two compounds was found between the signals assigned to C-12 and C-13 (5.6 ppm), and it was inferred that the structural difference of compound **2** and compound **1** occurs in this part of the molecule. In fact, the only plausible explanation for these observed differences is that compound **2** is an epimer of compound **1**, bearing the 12-hydroxyl group in a different configuration. Previously, several cases were reported in which the relative configuration of the 12-OH group of a *Holothuria* triterpene glycoside was determined based on NOESY correlations [3,7,8]. It was stated that, in the case of an *α*-configuration, a correlation of H-12 with a proton signal originating from the methyl group attached to C-20 (H_3_-21) was observed [3,7,8,84], and in general, this relative configuration is reported for this type of compound. When comparing the NOESY spectra obtained for compounds **1** and **2** (supplementary Figures S5 and S13), in the first case, a correlation was indeed observed between H-12 (4.53 ppm) and H_3_-21 (1.50 ppm), while no correlation was observed for the H-12 (5.31 ppm) and H_3_-21 (1.21 ppm) of compound **2**. Thus, these results are in line with the proposed structure of compound **2**, bearing a 12*β*-OH group, i.e., 12-*epi*-desholothurin B (Figure 5). To the best of our knowledge, this compound has not been reported before.

With regards to the other stereocenters, the same relative configurations are assigned to compounds **1** and **2,** as previously reported for holothurin B. This configuration is most commonly reported for the triterpene glycosides of the *Holothuria* genus in general [73,84,85].

### 3.3. Fatty Acid Profile of the H. atra Body Wall

The Me90 was analyzed by GC-MS, and this led to the identification of 11 fatty acids, including both saturated and unsaturated fatty acids (Table 4). Several fatty acids were previously reported in *H. atra* [36,46,87], while two of the identified fatty acids are reported herewith for the first time in *H. atra*.

### 3.4. α-Glucosidase Inhibition Assay

The α-glucosidase inhibition assay was conducted to determine the anti-diabetic potential of the *H. atra* body wall. Acarbose was used as a positive control (Table 5). In order to further study the activity of the Me90 fraction, commercially available fatty acids were also tested against α-glucosidase (Table 6). A previous study, conducted by Su and team, revealed that fatty acids are potent α-glucosidase inhibitors [43,44,56]. The IC_50_ values of the MG subfraction and the Me90 fraction did not differ significantly. The IC_50_ values of fatty acids (palmitoleic acid, arachidonic acid, and eicosapentaenoic acid) were significantly different to those of the acarbose.

## 4. Discussion

In this study, 11 triterpene glycoside compounds were tentatively identified in the sea cucumber *H. atra.* While the applied UPLC-HRMS analysis can provide information on the molecular weight of the saponin, and often of the sapogenin, a distinction between the triterpene glycosides with isomeric sapogenin moieties and those with glycosidic moieties is, in many cases, impossible. Therefore, for several of the identified signals, more than one possible identified compound was listed in Table 1.

In general, triterpene glycosides contain an aglycone which is either a 3β-hydroxyholost-7-ene or a 3β-hydroxyholost-9(11)-ene aglycone [88]. *Holothuria* triterpene glycosides contain an aglycone moiety with a holostanol skeleton. Puspitasari and co-workers grouped the sapogenins of the *Holothuria* triterpene glycoside into six types [32]. Such sapogenins are common sapogenins in the genus *Holothuria* [32,80,89]. Triterpene glycosides are also classified based on their types of side chain, including those with a tetrahydrofuran group and those with a linear side chain. With regards to the side chain, 25 types are commonly identified within the *Holothuria* genus. In this work, 11 types of triterpene glycosides were identified from the body walls of *H. atra* [32,89].

In this study, only one compound, calcigeroside B (Figure 3), contains a double bond between C-7 and C-8, while a 9(11)-double bond is present in all the other triterpenoid glycosides of *H. atra*. Despite calcigeroside B, nobiliside (II) or ananaside C (Figure 4) also has an atypical structure of triterpene glycosides, and this compound was previously identified from *H. nobilis* [90].

In this work, the glycosidic moieties were comprised of D-xylose, D-quinovose, D-glucose, and MeGlc sugars, attached to C-3 of the sapogenin, with xylose being the first sugar (Figure 2). Puspitasari et al. reported that there are approximately 20 types of sugar units attached to the *Holothuria* triterpene glycosides [32]. In addition, in this study, the glycosidic moiety comprised two or four monosaccharide units (Figure 2), and the first monosaccharide was linked to a sulfate group. However, desholothurin B (**1**) and 12-epi-desholothurin B (**2**) are exceptions, neither bearing a sulfate group. The sugar moieties bound to the aglycone are commonly reported with the β-configuration [32,80,89,91]. Leucospilatoside A, holothurin B, B1, B2, B3, and B4, echinoside B, nobiliside (II) (=ananaside C), and 24-dehydroechinoside B have two sugar units: D-xylose and D-quinovose [64,72,73,83,92]. The other detected sea cucumber saponins have four sugar residues, all in the following order: D-xylose, D-quinovose, D-glucose, and MeGlc. Calcigeroside B (Figure 3) is an atypical *Holothuria* saponin with a triple-branched glycosidic moiety, composed of D-xylose, D-quinovose, D-glucose, 3-*O*-methylxylose, and D-quinovose. The aglycone moiety of calcigeroside B is also atypical [32,37,89,93]. In total, two monosaccharides, namely D-xylose and D-quinovose, are also present in both purified compounds. Nobiliside II (=ananaside C) (Figure 4) is a sulfated triterpene glycoside with two sugar moieties: xylose and quinovose. Its chemical structure is defined as 3-*O*-{β-*D*-quinovopyranosyl-(1 → 2)-4′-*O*-sulfate-β-*D*-xylopyranosyl}-holoshillaside 18 (16)-lactone-22, 25-epoxy-9-ene-3β, 12α, 17α-triol sodium [90].

With respect to the nomenclature of the sea cucumber saponins, it is important to be aware of the fact that, in some cases, the same compound was named differently by different authors, while, in other cases, different compounds got the same name. Honey-Escandon stated that the chemical nomenclature of the triterpene glycosides could be inconsistent and homonyms occur [89]. In this case, holothurin A5 was identified in *H. atra* with the molecular formula C_54_H_83_O_28_SNa and a nominal mass of 1234 [37], while it was reported later with the same compound name, holothurin A5, but with the different molecular formula C_54_H_85_O_28_SNa and a nominal mass of 1236 [66]. Wu et al. reported ananaside C as a new compound from *Thelenota ananas* in 2007. However, a triterpene glycoside with the same formula was reported as nobiliside II from *H. nobilis* by Zhang in 2011 [89,90].

Triterpene glycosides such as holothurin B/B4, B1, B2, and B3, echinoside A and B, 24-dehydroechinoside A, calcigeroside B, and holothurin D were previously identified in *H. atra* [11,12,32,35,36,37]. Some other compounds have not been found in *H. atra* up until now, but were reported from other *Holothuria* species. Leucospilotasides A was previously isolated from the *H. leucospilota* body wall, which was collected in the South China Sea [64,72]. Other researchers reported the identification of this compound from the Vietnamese *H. edulis* body wall [66]. Leucospilotaside A was also identified in the viscera of *H. lessoni* [58]. Holothurin A1 and holothurin A4 were isolated from *H. floridana* and *H. grisea* [67]. Scabraside D was detected from *H. scabra* and *H. lessoni* [10,68,94]. 17-dehydroxyholothurin A and fuscocineroside B and C were isolated from *H. impatiens* and *H. fuscocinerea*, respectively [7,70]. Scabraside A, holothurin E, and fuscocineroside B and C were also detected in *H. lessoni* [58,59].

In this study, the sea cucumber saponins identified by means of UPLC-HRMS analysis were tentatively identified as sulfated saponins. In contrast, the two compounds isolated from the body wall of the *H. atra*: desholothurin B (**1**) and epi-desholothurin B (**2**), were not sulfated [84]. Van Dyck et al. [12] and Omran et al. [36] stated that *H. atra* contains only sulfated triterpene glycosides, such as echinoside A, echinoside B, and holothurins B/B4, B1, B2, and B3. However, according to Oleinikova and co-workers, desulfated holothurin B was detected in *H. atra* [86].

According to Ridzwan and co-workers, the body wall of the *H. atra* contained 57.04% of saturated fatty acids, 4.31% of monounsaturated fatty acids, and 38.64% of polyunsaturated fatty acids [46]. With regards to the lipid fraction of the *H. atra*, the saturated fatty acids were myristic acid, pentadecanoic acid, palmitic acid, stearic acid, arachidic acid, heneicosanoic acid, and behenic acid, while the other identified fatty acids were unsaturated. Myristic acid, palmitoleic acid, palmitic acid, stearic acid, arachidonic acid, eicosapentanoic acid, arachidic acid, behenic acid or docosanoic acid, and nervonic acid were previously reported [36,46], while nervonic acid and heneicosanoic acid are reported in *H. atra* for the first time. Arachidic acid was previously identified in *H. edulis* and *Bohadschia marmorata.* Nervonic acid was reported in *H. polii, H. edulis,* and *B. marmorata* [36].

As for the α-glucosidase inhibition, the MG subfraction was found to be more active than acarbose. The following compounds were tentatively identified in subfraction G: leucospilatoside A, holothurin B3 or 24-dehydroechinoside B, echinoside A (=holothurin A2), and 17-dehydroxyholothurin A (=fuscocineroside C), scabraside A, 24-dehydroechinoside A, or fuscocineroside B. Unfortunately, the amount of isolated saponins was not sufficient enough to allow for individual testing in the α-glucosidase inhibition assay, and therefore, the hypothesis that the isolated saponins present in the MG subfraction contributed to the observed inhibitory activity could not be confirmed.

The Me90 fraction also inhibited the α-glucosidase activity with an IC_50_ value of 0.158 ± 0.002 mg/mL. This fraction contains various fatty acids (Table 4). Besides saponins, fatty acids were also reported to be α-glucosidase inhibitors. Su and co-workers revealed that the IC_50_ values of palmitoleic acid and arachidonic acid against α-glucosidase were 0.265 ± 0.002 mM and 0.211 ± 0.002 mM, respectively [56]. It was reported that the body wall of the sea cucumber *S. japonicus* contains unsaturated fatty acids, namely 7(Z)-octadecenoic acid and 7(Z),10(Z)-octadecadienoic acid, and that these compounds inhibited α-glucosidase with IC_50_ values of 0.51 ± 0.02 µg/mL and 0.49 ± 0.05 µg/mL, respectively [43]. 1,3-Dipalmitolein and cis-9-octadecenoic acid, purified from *S. japonicas*, also showed α-glucosidase inhibitory activity, with IC_50_ values of 4.45 µM and 14.87 µM, respectively [44].

The inhibition of α-glucosidase activity in the small intestine is one strategy to suppress blood glucose levels. α-glucosidase, located on the brush-border of the small intestine, plays a pivotal role in carbohydrate digestion by hydrolyzing starch into monosaccharides (glucose). By inhibiting the α-glucosidase activity, carbohydrate digestion and glucose production can be delayed. Therefore, less glucose will be absorbed into the blood stream, which can reduce hyperglycemia [47]. This study revealed that some lipophilic and hydrophilic fractions of *H. atra* body walls can inhibit α-glucosidase. These fractions were found to contain several fatty acids and triterpene glycosides. Further studies will need to be conducted to determine the specific inhibitory activity of these compounds and to assess whether *H. atra* could play a role in the management of glucose levels in diabetic patients.

## 5. Conclusions

In conclusion, the phytochemical profile of *H. atra* body walls was investigated and 13 triterpene glycosides and 11 fatty acids were (tentatively) identified. The saponin 12-*epi*-desholothurin B (**2**) was purified and identified for the first time. It was found that several fractions of *H. atra* body walls showed α-glucosidase inhibitory activity.

## Figures and Tables

**Table 1:** Tentatively identified triterpene glycosides from *H. atra* based on UPLC-HRMS analysis in ESI^-^ mode.

Compound Number	Compound Name	RT (min)	Molecular Formula	Nominal Mass	Ion	Measured *m*/*z*	Error (ppm)	Fragmentation Pattern	Reference	Sample
1	17-hydroxyfuscocineroside B (=scabraside B) * or 25-hydroxyfuscocineroside B *	7.56	C_54_H_85_O_27_Sna	1220	[M-Na]^-^	1197.4982	−1.67	875.3718 [M-Na-322 (MeGlc+Qui)]^-^	[58,59,60,61,62,63]	c, f
2	Leucospilotaside A *	7.87	C_41_H_63_O_18_Sna	898	[M-Na]-	875.371	4.66	729.3176 [M-Na-146(Qui)]^-^ 755.2563 [M-Na-120(NaHSO_4_)]^-^	[58,64]	c, d, e, f, g
3	Holothurin A3 * or Holothurin A5 (II)*	8.53	C_54_H_85_O_28_Sna	1236	[M-Na]^-^	1213.4944	−0.66	875.3729 [M-Na-338 (MeGlc+Glc)]^-^	[65,66]	d
4	Holothurin A1 *, or Holothurin A4 * or Scabraside D *	9.06	C_54_H_87_O_27_Sna	1222	[M-Na]^-^	1199.5132	−1.66	861.3885 [M-Na-338 (MeGlc+Glc)]^-^ 843.3527 [M-Na-356 (MeGlc+Glc+H_2_O)]^-^	[58,65,67,68,69]	d, f
5	Calcigeroside B	9.59	C_54_H_83_O_27_Sna	1218	[M-Na]-	1195.486	1.51	Not observed	[37]	d, f
6	17-dehydroxyholothurin A * (=fuscocineroside C *), or Scabraside A *, or 24-dehydroechinoside A *, or Fuscocineroside B *	9.94	C_54_H_85_O_26_Sna	1204	[M-Na]-	1181.5032	−1.69	Not observed	[7,11,37,58,60,61,68,70,71]	e, g
7	Holothurin B2	10.27	C_41_H_65_O_17_Sna	884	[M-Na]^-^	861.3938	−0.46	715.7407 [M-Na-146(Qui)]^-^	[72,73]	c, f, g
8	Holothurin B, or Holothurin B4, or Nobiliside II (=ananaside C) *	10.70	C_41_H_63_O_17_Sna	882	[M-Na]-	859.3801	1.75	713.3293 [M-Na-146(Qui)]^-^	[11,12,37,58,66,73,74,75,76,77,78]	a, b, c, d, f, g
9	Echinoside A (=Holothurin A2)	10.89	C_54_H_87_O_26_Sna	1206	[M-Na]-	1183.5178	−2.03	1165.5055 [M-Na-18(H_2_O)]^-^	[60,71,76,79,80,81]	d, e, f
10	Holothurin B3 or 24-dehydroechinoside B *	11.02	C_41_H_63_O_16_Sna	866	[M-Na]-	843.3819	−2.13	697.3291 [M-Na-146(Qui)]-	[12,73,74,82]	c, d, e, f, g
11	Echinoside B (=Holothurin B1)	12.49	C_41_H_65_O_16_Sna	868	[M-Na]-	845.3994	0.11	827.3956 [M-Na-18(H_2_O)]^-^ 695.4776 [M-Na-150(Xyl)]^-^ 725.5139 [M-Na-120(NaHSO4)]^-^	[12,36,67,72,77,78,83]	c, d, e, f, g

* Compounds identified for the first time in *H. atra.* (a) subfraction MB, (b) subfraction MC, (c) subfraction ME, (d) subfraction MF, (e) subfraction MG, (f) Bu fraction, and (g) Me fraction.

**Table 2:** Molecular structure of triterpene glycosides identified from *H. atra* body wall.

Compound Number	Compound Name	Sapogenin*	R1 *	R2 *	R3 *
1	17-hydroxyfuscocineroside B (=scabraside B) or	A	OH	8	b
	25-hydroxyfuscocineroside B	A	H	9	b
2	Leucospilotaside A	A	OH	9	a
	Holothurin A3 or	A	OH	9	b
3	Holothurin A5	A	OH	11	b
4	Holothurin A1 or	A	OH	5	b
	Holothurin A4 or	A	OH	6	b
	Scabraside D	A	OH	7	b
5	Calcigeroside B	Atypical			
6	17-dehydroxyholothurin A (=fuscocineroside C) or	A	H	1	b
	Scabraside A or	A	OH	4	b
	24-dehydroechinoside A or	A	OH	3	b
	Fuscocineroside B	A	H	1	b
7	Holothurin B2	A	OH	5	a
8	Holothurin B, or	A	OH	1	a
	Holothurin B4, or	A	OH	10	a
	Nobiliside II (=ananaside C)	Atypical		1	a
9	Echinoside A (=Holothurin A2)	A	OH	2	b
10	Holothurin B3 or	A	H	1	a
	24-dehydroechinoside B	A	OH	3	a
11	Echinoside B (Holothurin B1)	A	OH	2	a

* Corresponding substructures of sapogenin, R1, R2, and R3 are shown in Figure 2.

**Table 3:** ^13^C and ^1^H-NMR chemical shifts (integration, multiplicity, J(Hz)) of desholothurin B (**1**) in methanol-*d*_4_ and 12-epi-desholothurin B (**2**) in pyridine-*d*_5_.

	1			2		
	δ ^13^C (ppm)	δ^1^H (ppm)		δ ^13^C (ppm)	δ ^1^H (ppm)	
1	37.3	1.85/1.51	(1H */1H *)	36.5	1.71/1.47	(1H */1H *)
2	27.7	1.97/1.79	(1H */1H *)	27.2	2.18/1.93	(1H */1H *)
3	90.3	3.13	(1H, d, 11.6)	89.0	3.24	(1H *)
4	40.8			40.3		
5	53.8	0.98	(1H, d, 11.2)	53.3	0.98	(1H, d, 11.7)
6	22.0	1.77/1.57	(1H */1H *)	21.4	1.72/1.59	(1H */1H *)
7	29.0	1.78/1.46	(1H */1H *)	27.7	1.70	(2H *)
8	41.9	3.01	(1H *)	40.8	3.26	(1H *)
9	155.6			151.2		
10	40.6			39.5		
11	115.4	5.38	(1H, d, 4.8)	119.5	5.62	(1H, br s)
12	72.5	4.53	(1H *)	66.9	5.25	(1H, br s)
13	59.7			65.3		
14	46.5			48.7		
15	37.5	1.79/1.16	(1H */1H *)	37.0	1.75/1.40	(1H */1H *)
16	35.9	2.80/2.07	(1H, dd, 15.1; 8.5/1H *)	37.3	3.11/2.35	(1H, dd, 14.5;7.2/1H, m)
17	90.5			87.4		
18	176.2			N.O.		
19	22.8	1.15	(3H, s)	22.0	1.40	(3H, s)
20	87.6			87.4		
21	18.7	1.50	(3H, s)	16.9	2.04	(3H, s)
22	81.5	4.20	(1H, t, 7.4)	81.7	4.27	(1H *)
23	28.6	2.04	(2H*)	28.4	2.00	(2H*)
24	39.2	1.78	(2H*)	38.6	1.56	(2H*)
25	82.9			81.5		
26	28.9	1.30	(3H, s)	28.6	1.02	(3H, s)
27	27.6	1.25	(3H, s)	27.7	1.13	(3H, s)
28	17.1	0.92	(3H, s)	16.9	2.04	(3H, s)
29	28.6	1.09	(3H, s)	28.2	1.31	(3H, s)
30	20.4	1.31	(3H, s)	19.9	1.37	(3H, s)
1’	106.0	4.42	(1H, d, 6.8)	105.8	4.78	(1H, d, 7.3)
2’	83.0	3.47	(1H *)	84.1	4.08	(1H *)
3’	77.8	3.52	(1H *)	78.2	4.16	(1H *)
4’	71.1	3.52	(1H *)	71.0	4.14	(1H *)
5’	66.4	3.86/3.21	(1H, dd, 11.5; 4.0/1H *)	66.8	4.24/3.61	(1H */1H, t, 10.1)
1’’	105.6	4.55	(1H)	106.4	5.18	(1H *)
2’’	76.8	3.25	(1H *)	77.3	4.08	(1H *)
3’’	77.5	3.33	(1H *)	77.9	4.10	(1H *)
4’’	77.0	2.99	(1H, t, 9.0)	76.8	3.70	(1H *)
5’’	73.7	3.29	(1H *)	73.6	3.78	(1H *)
6’’	18.2	1.27	(3H, s)	18.8	1.65	(3H, s)

* Peaks overlapping and/or multiplicity not clear.

**Table 4:** Fatty acids in MeOH 90% extract of *H. atra* body wall as identified by GC-MS.

Compound	Molecular Formula
Myristic acid	C_14_H_28_O_2_/C_14_:0
Pentadecanoic acid	C_15_H_30_O_2_/C_15_:0
Palmitoleic acid	C_16_H_30_O_2_/C_16_:1 ω-7
Palmitic acid	C_16_H_32_O_2_/C_16_:0
Stearic acid	C_18_H_36_O_2_/C_18_:0
Arachidonic acid	C_20_H_32_O_2_/C_20_:4n-6 ω-6
Eicosapentaenoic acid	C_20_H_30_O_2_/C_20_:5n-3 ω-3
Arachidic acid	C_20_H_40_O_2_/C20:0
Heneicosanoic acid *	C_21_H_42_O_2_/C_21_:0
Behenic acid	C_22_H_44_O_2_/C_22_:0
Nervonic acid *	C_24_H_46_O_2_/C_24_:1n-9 ω9

* Compounds identified for the first time in *H. atra.*

**Table 5:** IC_50_ values of extracts of *H. atra* against α-glucosidase.

Sample	IC_50_ (mg/mL)
Crude extract	>20
MB subfraction	>10
MC subfraction	>10
ME subfraction	0.469 ± 0.029 ^c,d^
MF subfraction	1.599 ± 0.361 ^b^
MG subfraction	0.181 ± 0.006 ^d^
Hex fraction	>20
Me fraction	>20
Bu fraction	0.779 ± 0.066 ^c^
Aq fraction	> 20
Me90 fraction	0.158 ± 0.002 ^d^
Acarbose	2.340 ± 0.044 ^a^

Different letters express significant differences between fractions (Tukey’s *p* < 0.05).

**Table 6:** IC_50_ values of fatty acids against α-glucosidase.

Sample	IC_50_ (mM)
Palmitoleic acid	0.320 ± 0.032 ^b^
Arachidonic acid	0.596 ± 0.044 ^b^
Eicosapentaenoic acid	0.541 ± 0.039 ^b^
Acarbose	3.533 ± 0.462 ^a^

Different letters express significant differences between fractions (Tukey’s *p* < 0.05).

## Data Availability

Not applicable.

## Supplementary Materials

The following supporting information can be downloaded at: https://www.mdpi.com/article/10.3390/nu15041033/s1, Figure S1: 1H NMR spectrum of compound 1 (desholothurin B) in CD3OD; Figure S2: COSY spectrum of compound 1 (desholothurin B) in CD3OD; Figure S3: HSQC spectrum of compound 1 (desholothurin B) in CD3OD; Figure S4: HMBC spectrum of compound 1 (desholothurin B) in CD3OD; Figure S5: NOESY spectrum of compound 1 (desholothurin B) in CD3OD; Figure S6: 13C spectrum of compound 1 (desholothurin B) in CD3OD; Figure S7: 13C DEPT 90 spectrum of compound 1 (desholothurin B) in CD3OD; Figure S8: 13C DEPT 135 spectrum of compound 1 (desholothurin B) in CD3OD; Figure S9: 1H spectrum of compound 2 (12-epi-desholothurin B) in pyridine-d5; Figure S10: COSY spectrum of compound 2 (12-epi-desholothurin B) in pyridine-d5; Figure S11: HSQC spectrum of compound 2 (12-epi-desholothurin B) in pyridine-d5; Figure S12: HMBC spectrum of compound 2 (12-epi-desholothurin B) in pyridine-d5; Figure S13: NOESY spectrum of compound 2 (12-epi-desholothurin B) in pyridine-d5; Figure S14: ESI+ HRMS spectrum of compound 1 (desholothurin B); Figure S15: ESI+ HRMS spectrum of compound 2 (12-epi-desholothurin B); Figure S16: ESI+ HRMS spectrum of fragment ions of compound 1 (desholothurin B); Figure S17: ESI+ HRMS spectrum of fragment ions of compound 2 (12-epi-desholothurin B).
