# Supplementary material for: Saponin and Fatty Acid Profiling of the Sea Cucumber Holothuria atra, α-Glucosidase Inhibitory Activity and the Identification of a Novel Triterpene Glycoside
Source: Nutrients. 2023 Feb 19;15(4):1033. doi: 10.3390/nu15041033 (PMC9960930; doi:10.3390/nu15041033)
Supplement: Supplementary file 1 [file nutrients-15-01033-s001.zip › nutrients-2144703-supplementary.pdf]

nutrients

MDPI

Supplementary Materials

Saponin and Fatty Acid Profiling of the Sea Cucumber *Holothuria atra*, α-Glucosidase Inhibitory Activity and the Identification of a Novel Triterpene Glycoside

Yunita Eka Puspitasari [1,2,3,*], Emmy Tuenter [1], Kenn Foubert [1], Herawati Herawati [4], Anik Martinah Hariati [5], Aulanni'am Aulanni'am [6], Luc Pieters [1], Tess De Bruyne [1] and Nina Hermans [1,*]

SUPPLEMENTARY MATERIALS

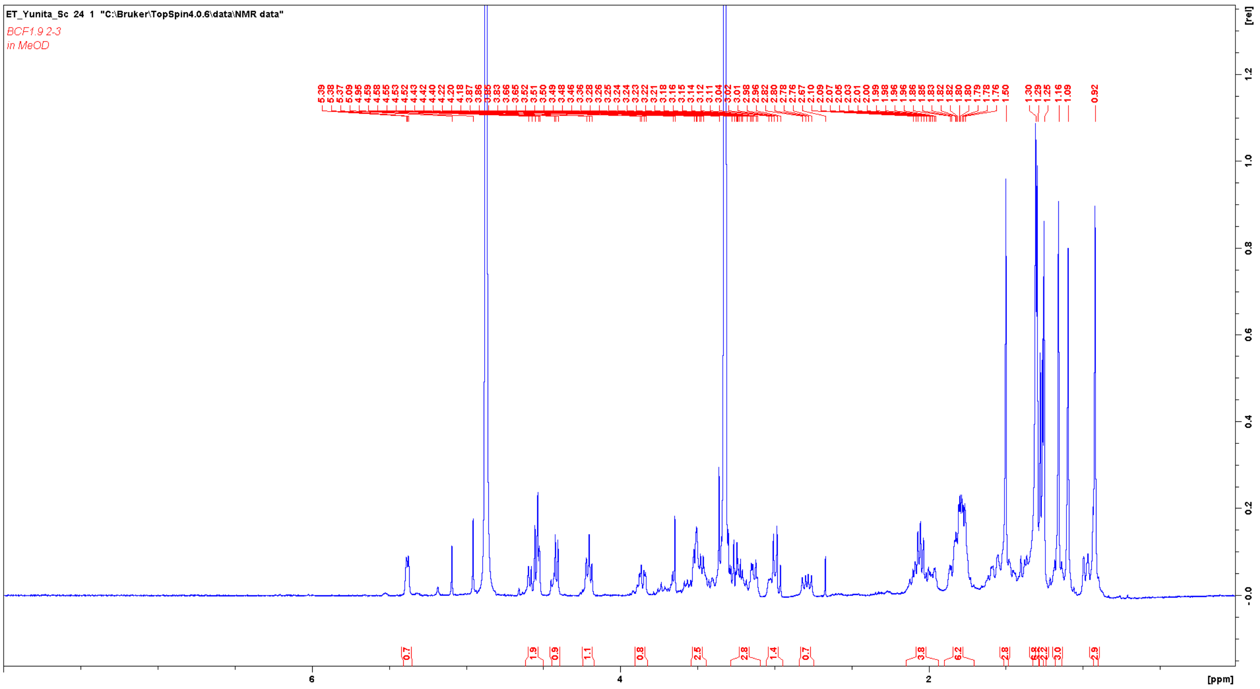

Figure S1. ^{1}H NMR spectrum of compound **1** (desholothurin B) in CD_3OD.

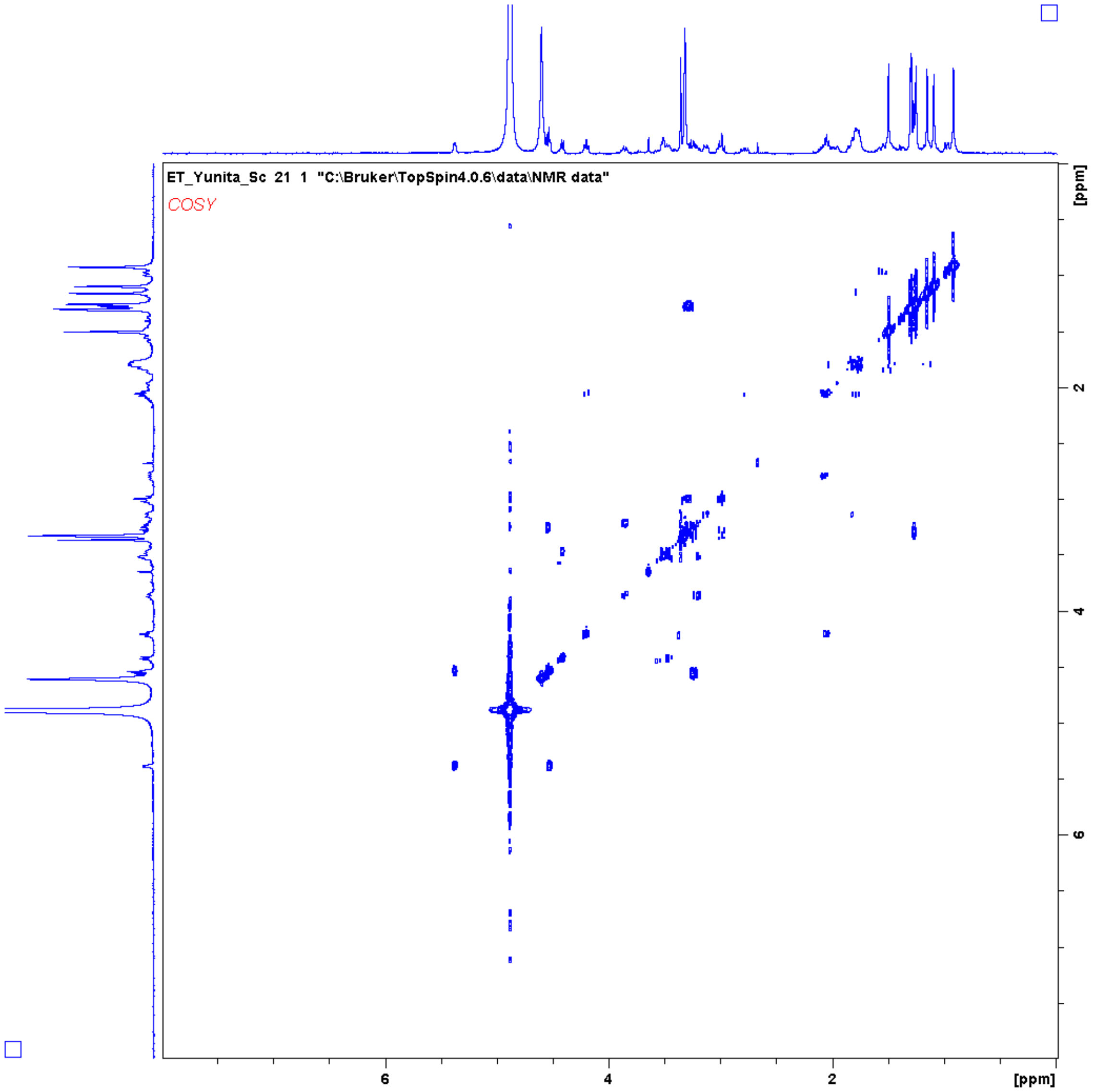

Figure S2. COSY spectrum of compound **1** (desholothurin B) in CD_3OD.

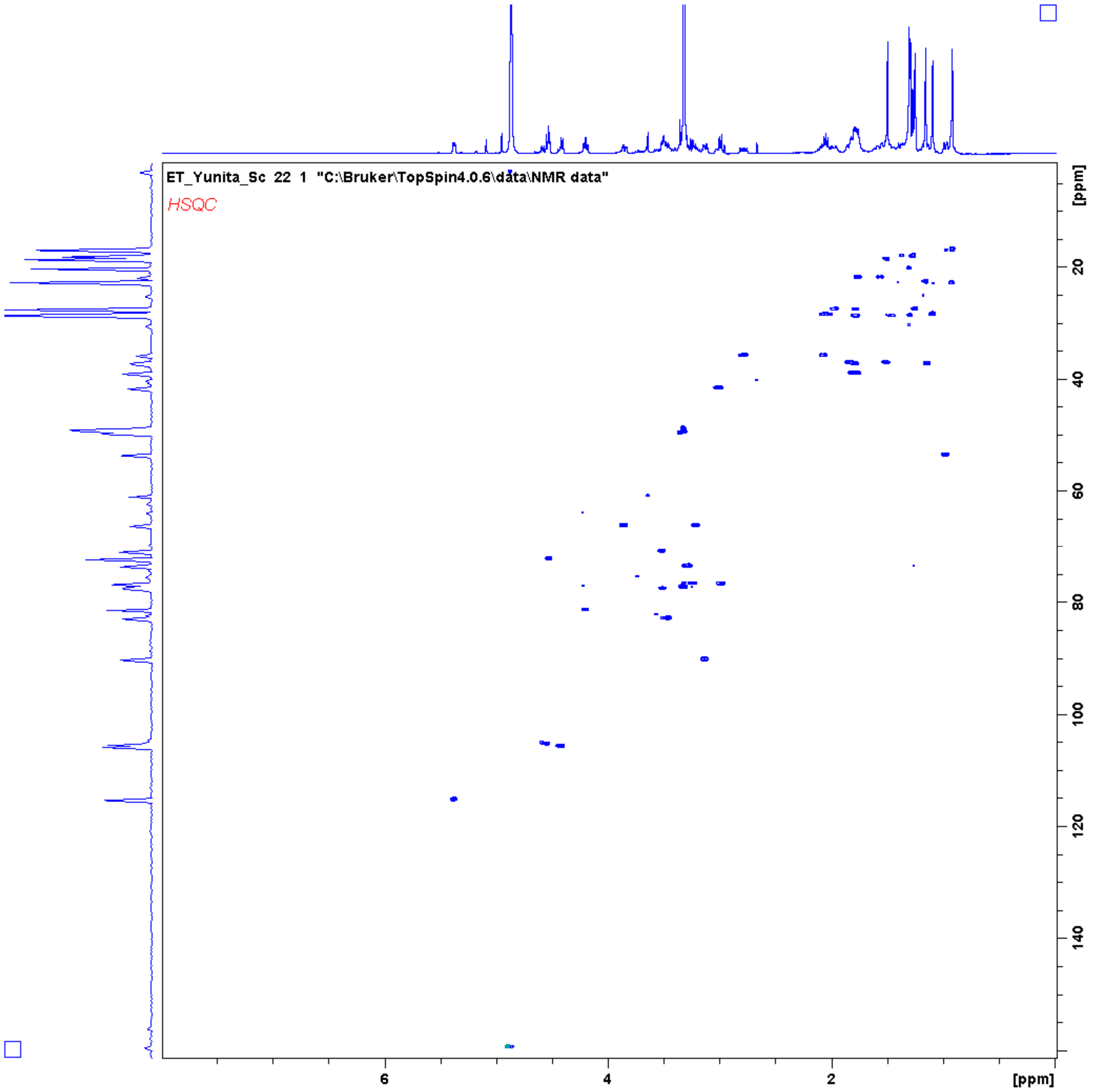

Figure S3. HSQC spectrum of compound **1** (desholothurin B) in CD_3OD.

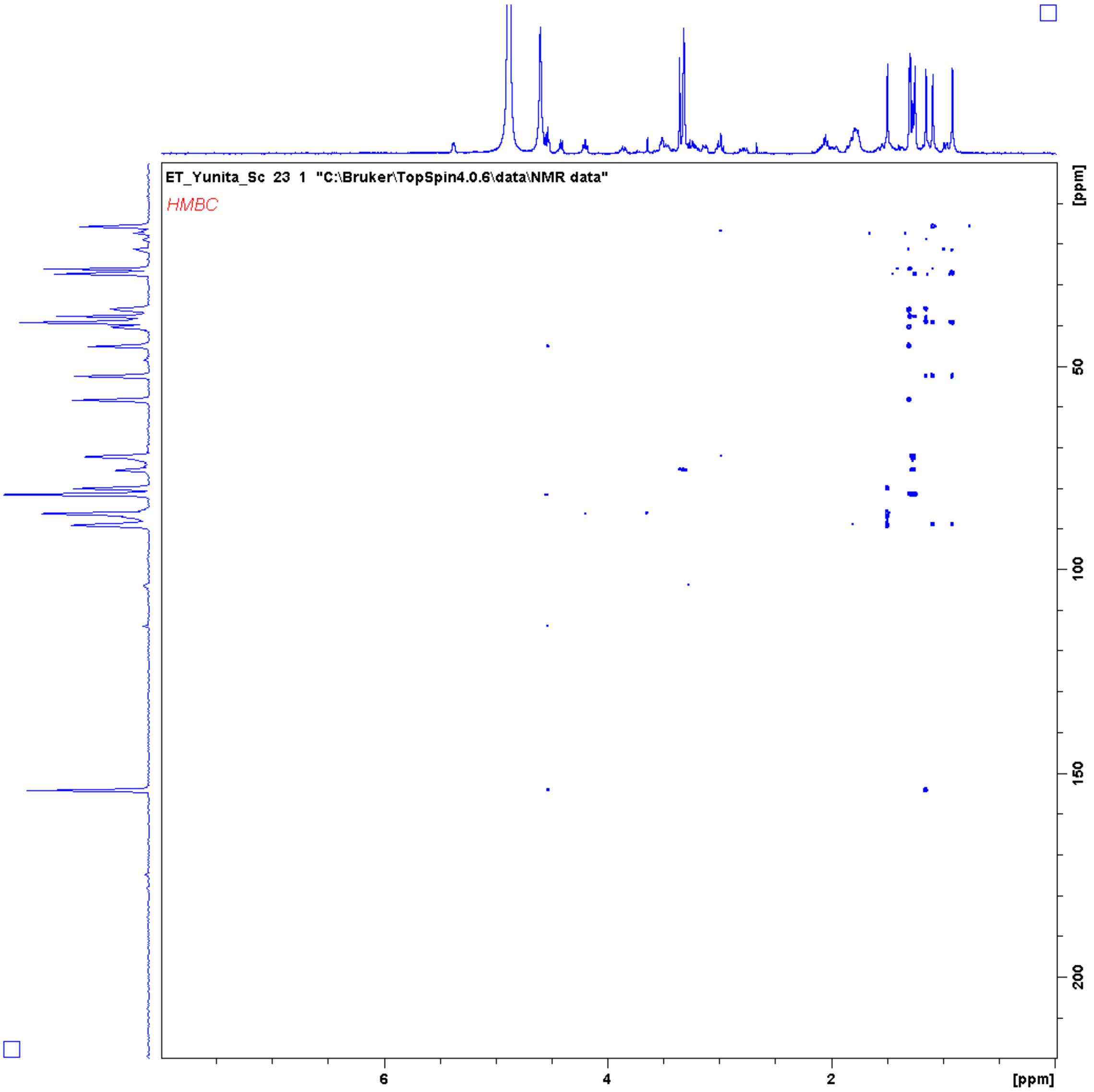

Figure S4. HMBC spectrum of compound **1** (desholothurin B) in CD_3OD.

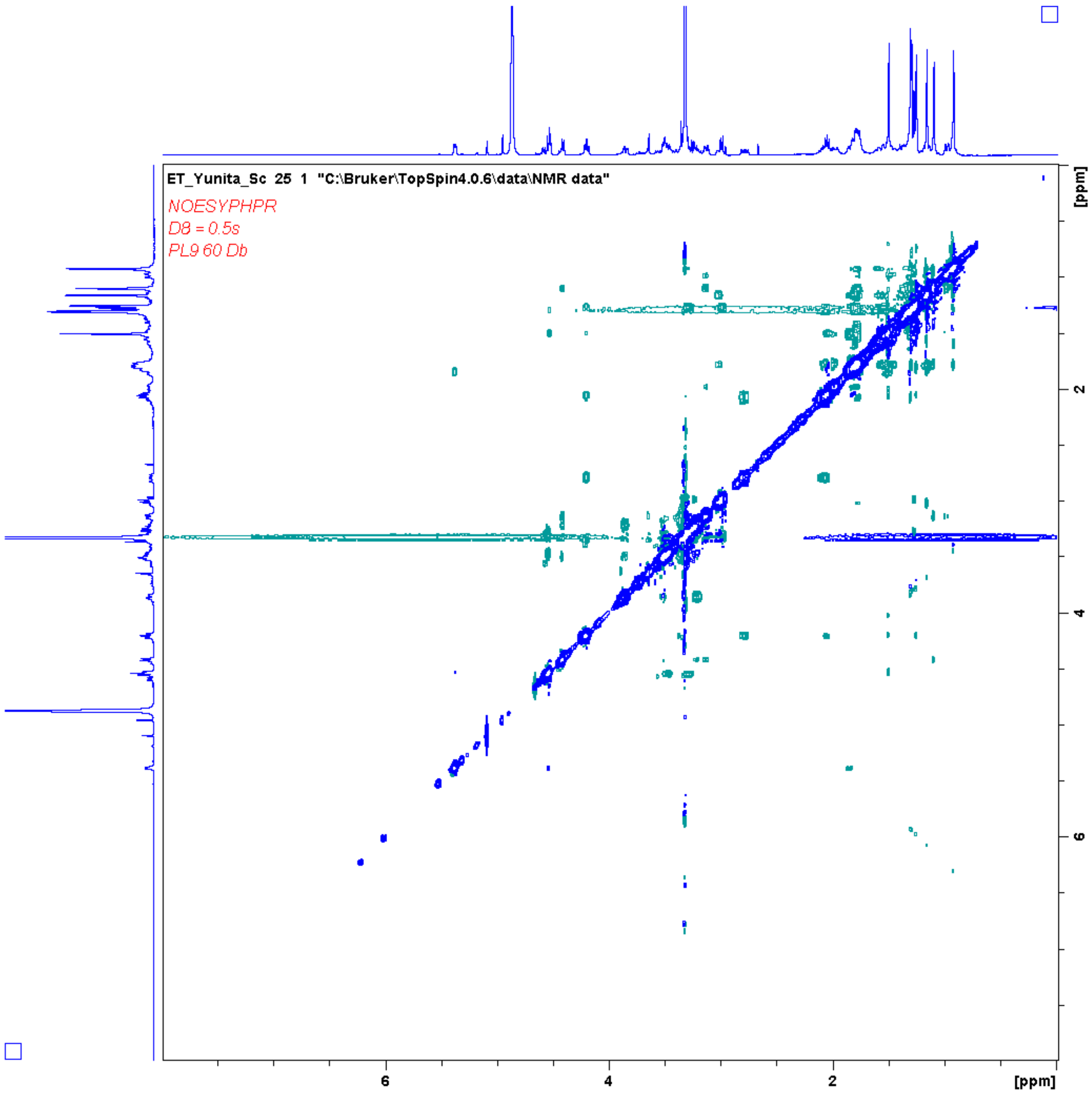

Figure S5. NOESY spectrum of compound **1** (desholothurin B) in CD_3OD

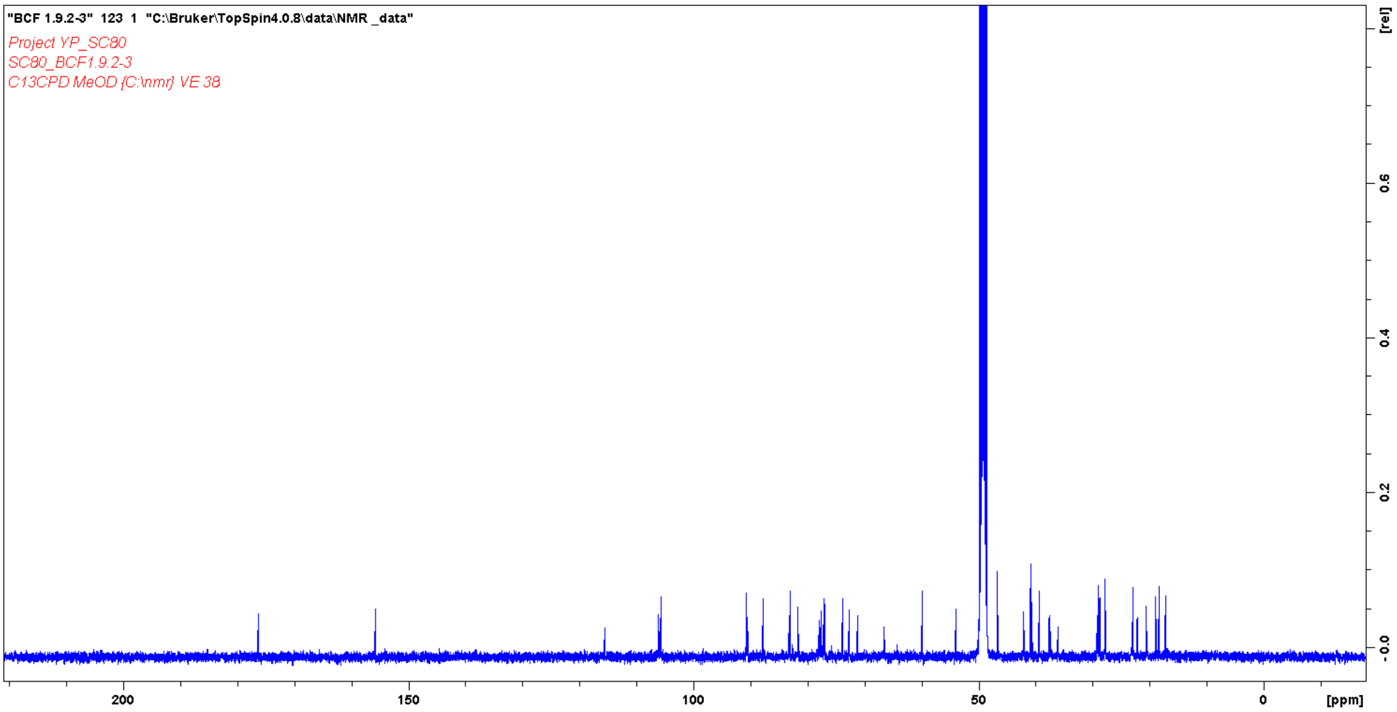

Figure S6. ^{13}C spectrum of compound **1** (desholothurin B) in CD_3OD.

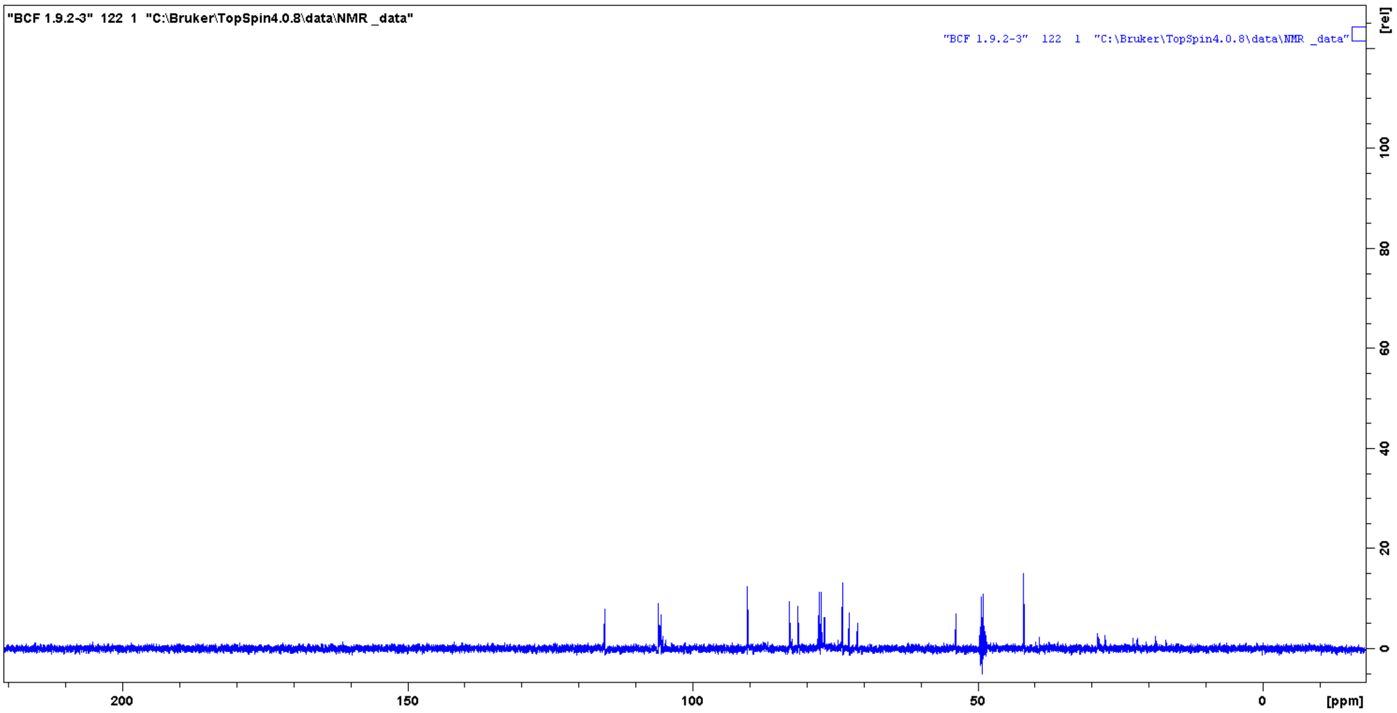

Figure S7. ^{13}C DEPT 90 spectrum of compound **1** (desholothurin B) in CD_3OD.

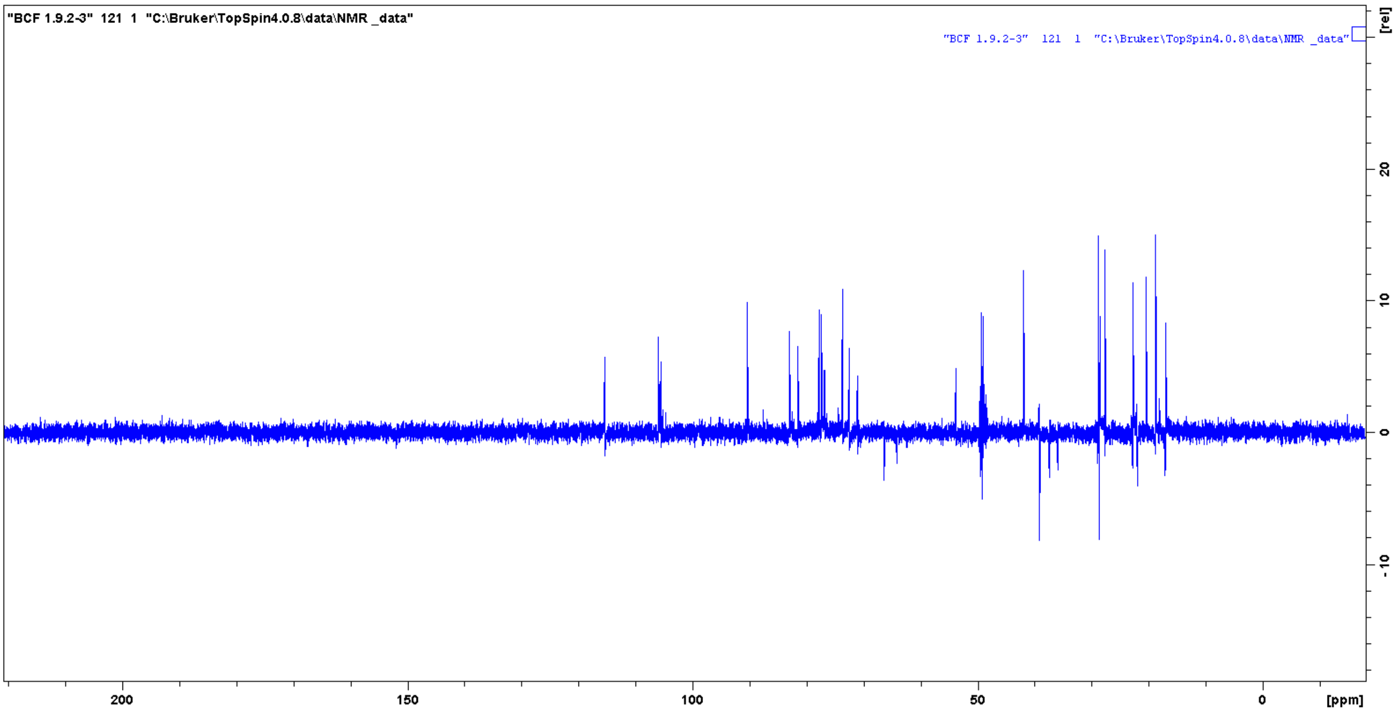

Figure S8. ^{13}C DEPT 135 spectrum of compound **1** (desholothurin B) in CD_3OD.

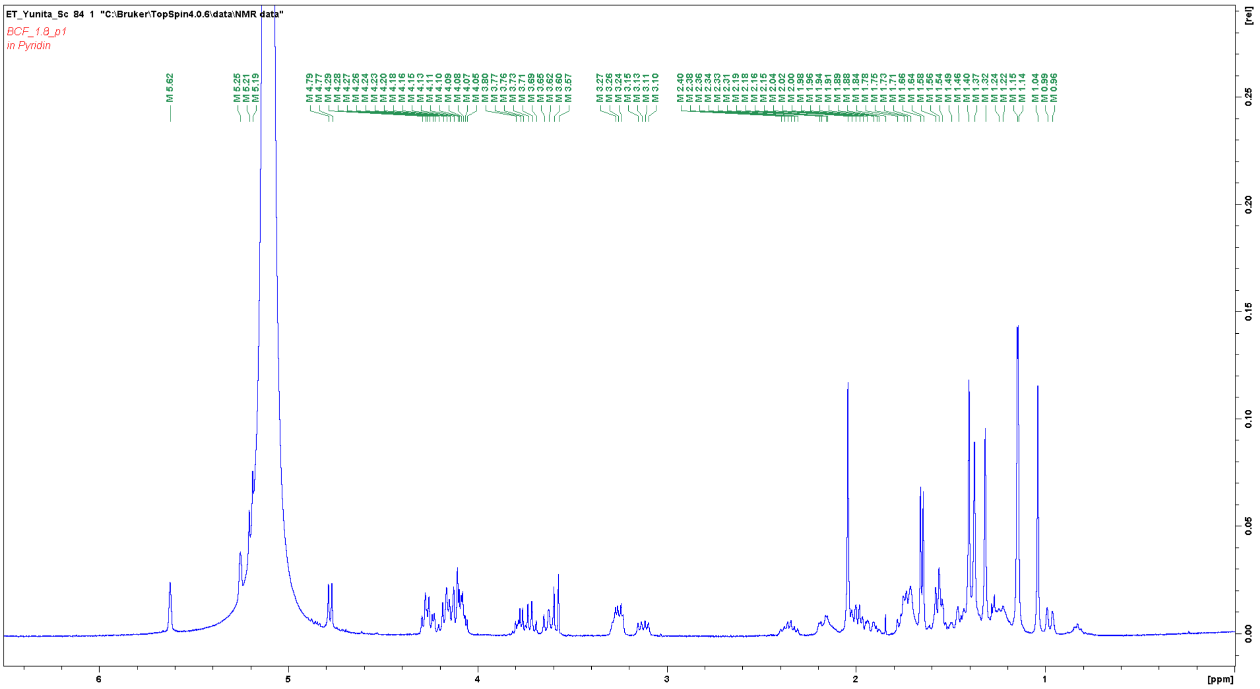

Figure S9. ^{1}H spectrum of compound **2** (12-*epi*-desholothurin B) in pyridine-d_5.

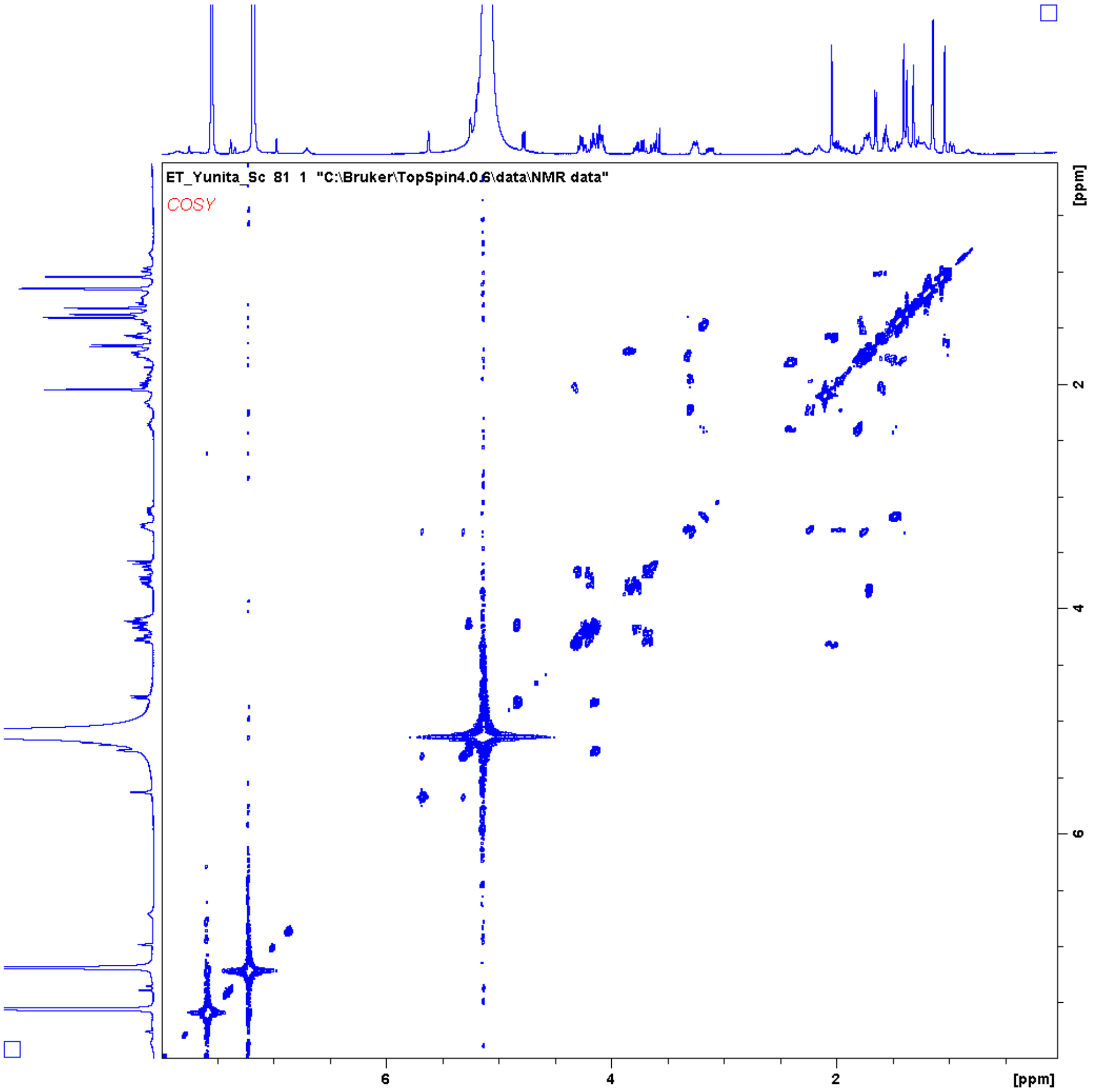

Figure S10. COSY spectrum of compound **2** (12-*epi*-desholothurin B) in pyridine-d_5.

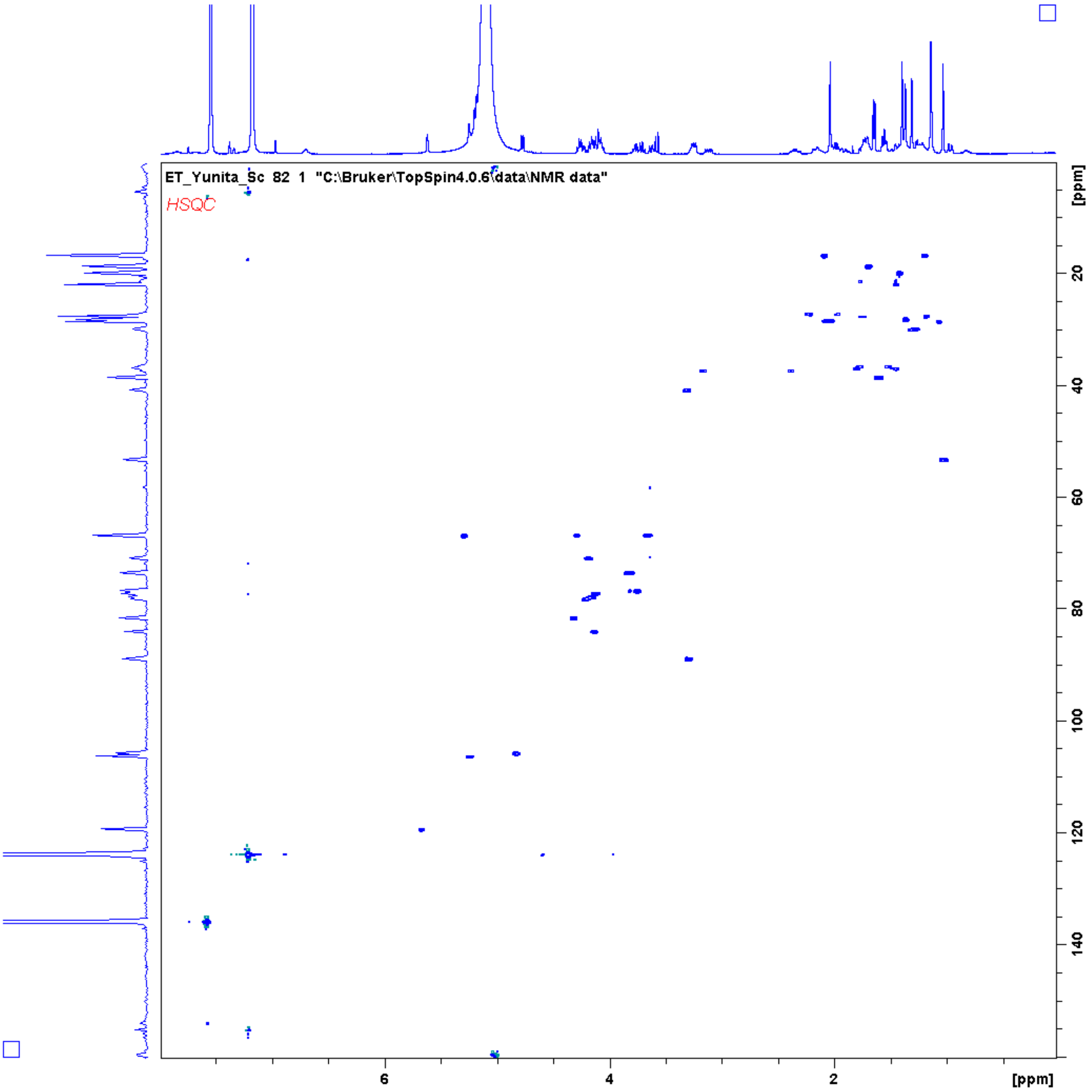

Figure S11. HSQC spectrum of compound **2** (12-*epi*-desholothurin B) in pyridine-*d*5.

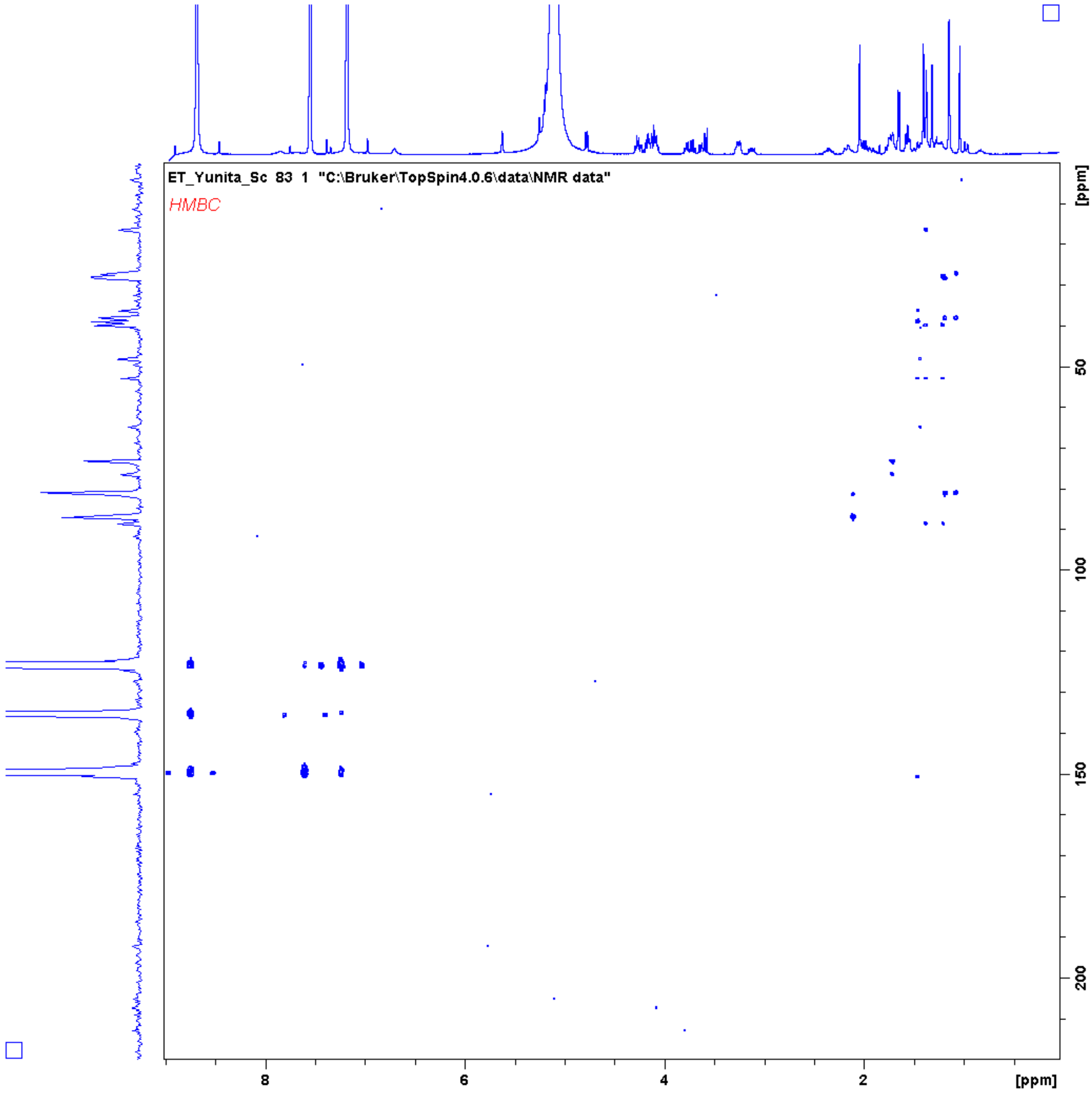

Figure S12. HMBC spectrum of compound **2** (12-*epi*-desholothurin B) in pyridine-d_5.

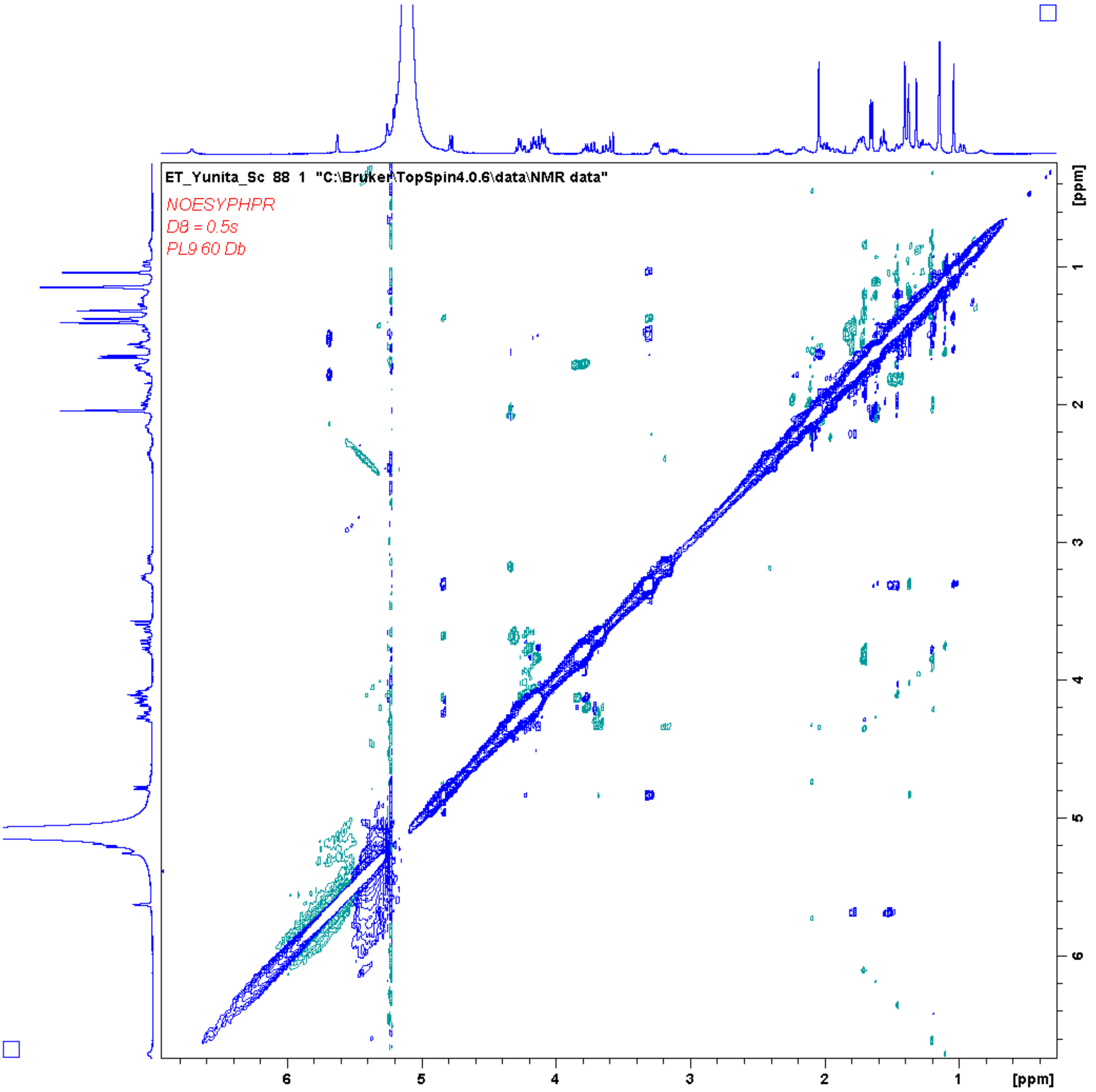

Figure S13. NOESY spectrum of compound **2** (12-*epi*-desholothurin B) in pyridine-d_5.

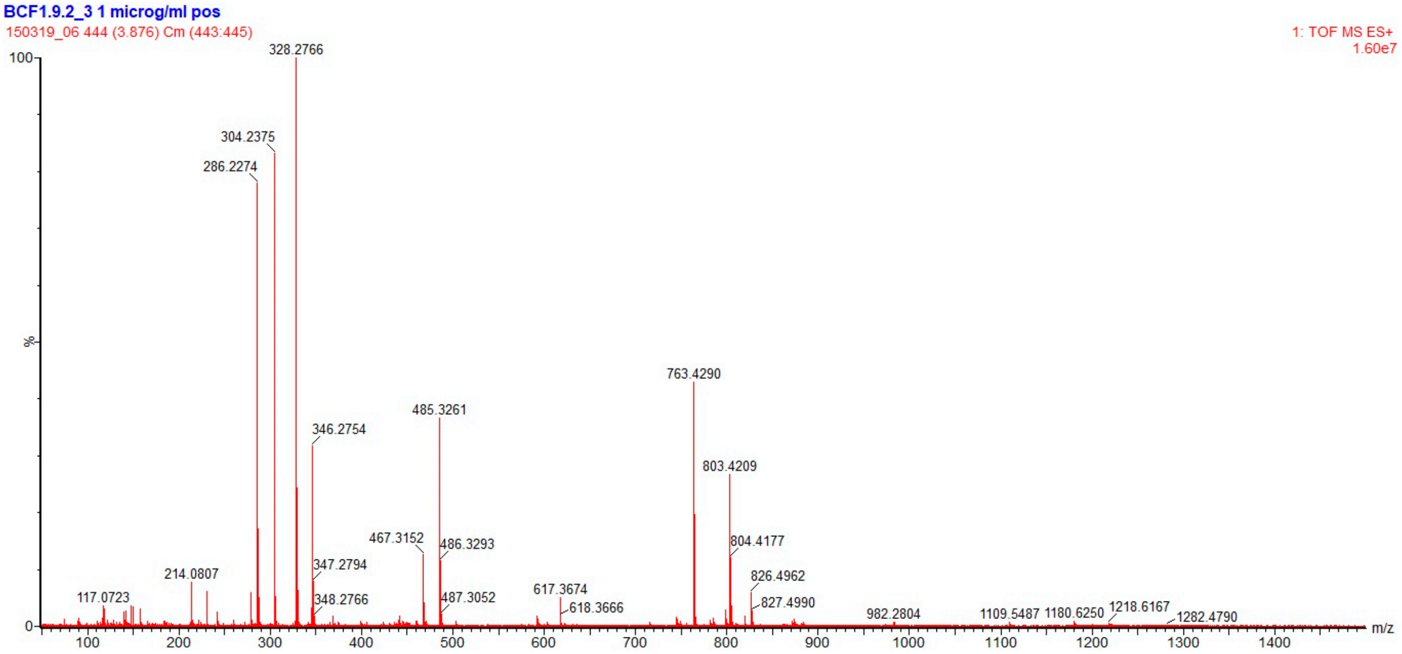

Figure S14. ESI+ HRMS spectrum of compound **1** (desholothurin B).

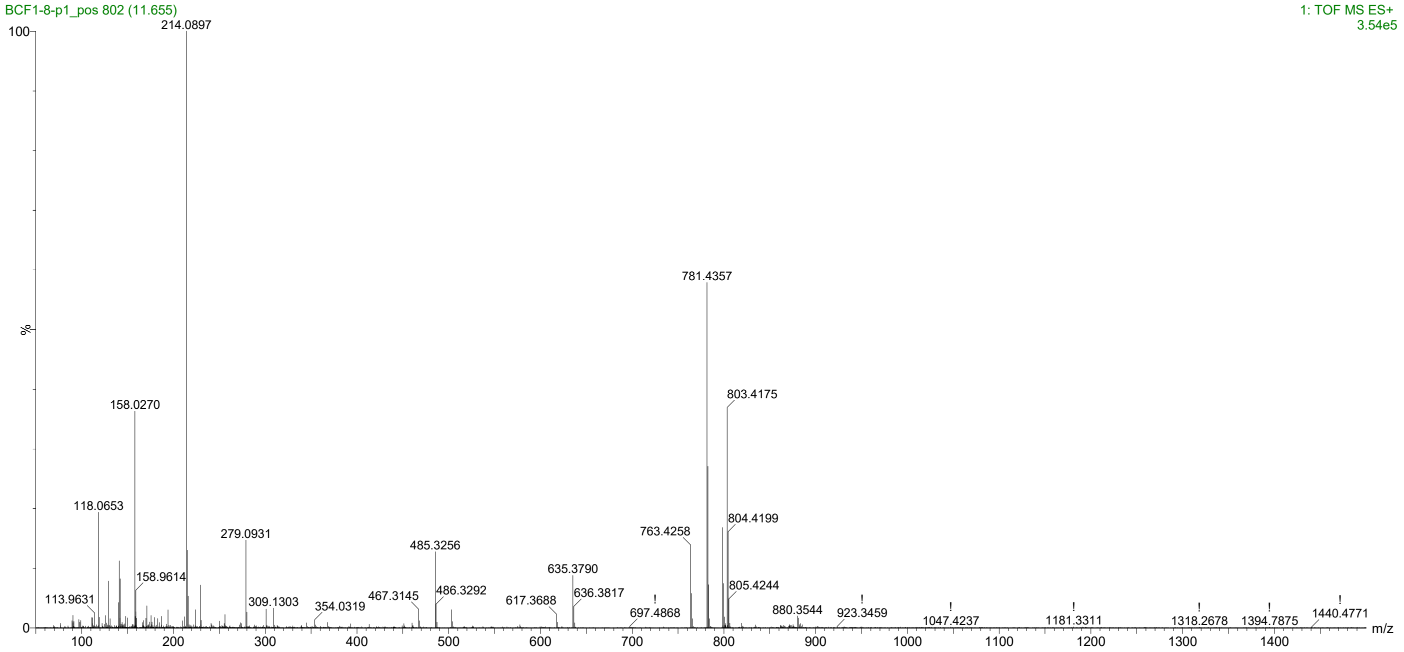

Figure S15. ESI+ HRMS spectrum of compound **2** (12-*epi*-desholothurin B).

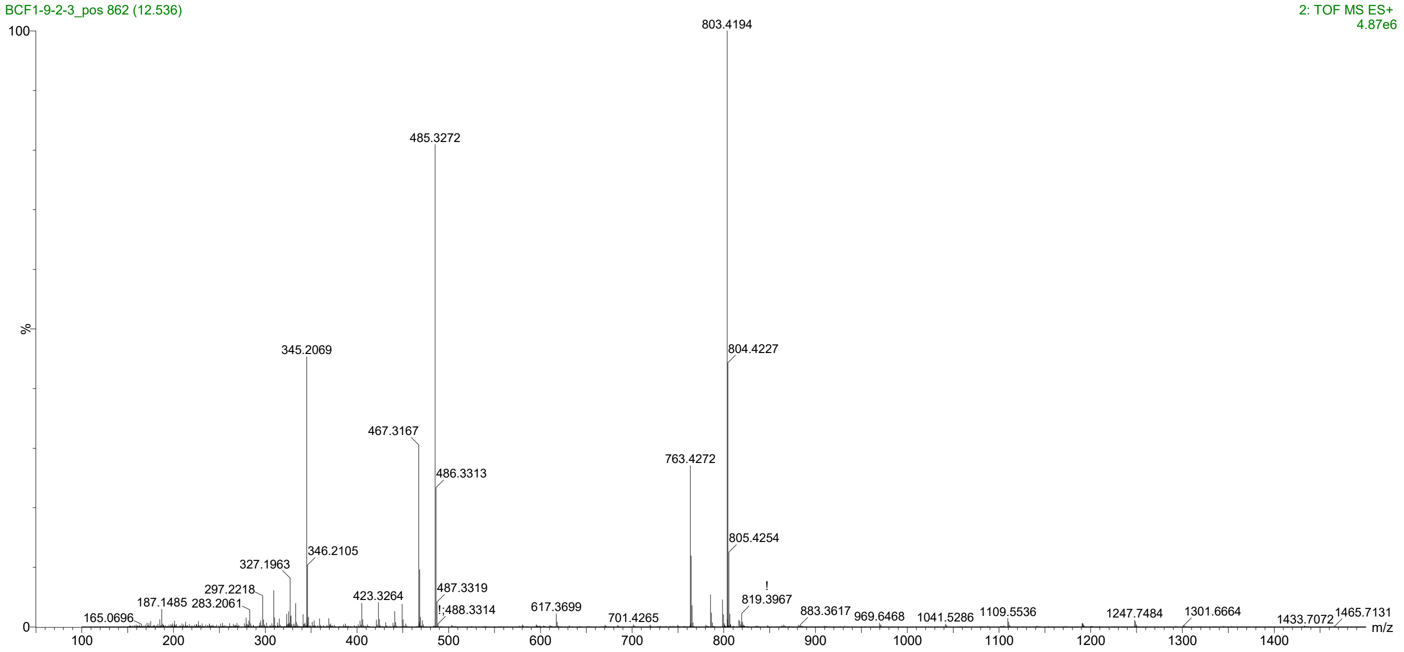

Figure S16. ESI+ HRMS spectrum of fragment ions of compound **1** (desholothurin B).

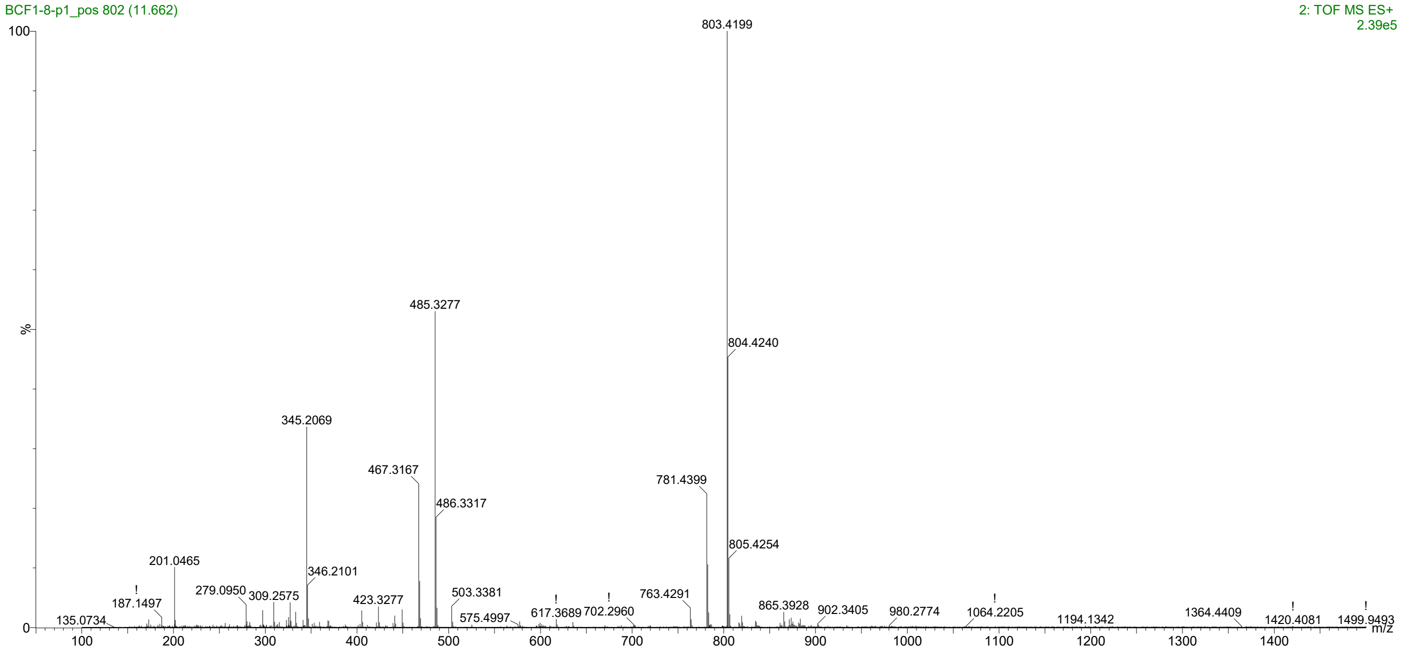

Figure S17. ESI+ HRMS spectrum of fragment ions of compound **2** (12-*epi*-desholothurin B).
